# Genome-wide chemical mutagenesis screens allow unbiased saturation of the cancer genome and identification of drug resistance mutations

**DOI:** 10.1101/gr.213546.116

**Published:** 2017-04

**Authors:** Jonathan S. Brammeld, Mia Petljak, Inigo Martincorena, Steven P. Williams, Luz Garcia Alonso, Alba Dalmases, Beatriz Bellosillo, Carla Daniela Robles-Espinoza, Stacey Price, Syd Barthorpe, Patrick Tarpey, Constantine Alifrangis, Graham Bignell, Joana Vidal, Jamie Young, Lucy Stebbings, Kathryn Beal, Michael R. Stratton, Julio Saez-Rodriguez, Mathew Garnett, Clara Montagut, Francesco Iorio, Ultan McDermott

**Affiliations:** 1 Wellcome Trust Sanger Institute, Hinxton CB10 1SA, United Kingdom;; 2 European Molecular Biology Laboratory – European Bioinformatics Institute, Cambridge CB10 1SA, United Kingdom;; 3 Pathology Department, Hospital del Mar, 08003 Barcelona, Spain;; 4 Laboratorio Internacional de Investigación sobre el Genoma Humano, Universidad Nacional Autónoma de México, Campus Juriquilla, Santiago de Querétaro 76230, Mexico;; 5 Cancer Research Program, FIMIM and Medical Oncology Department, Hospital del Mar, 08003 Barcelona, Spain;; 6 RWTH Aachen University Hospital, 52062 Aachen, Germany

## Abstract

Drug resistance is an almost inevitable consequence of cancer therapy and ultimately proves fatal for the majority of patients. In many cases, this is the consequence of specific gene mutations that have the potential to be targeted to resensitize the tumor. The ability to uniformly saturate the genome with point mutations without chromosome or nucleotide sequence context bias would open the door to identify all putative drug resistance mutations in cancer models. Here, we describe such a method for elucidating drug resistance mechanisms using genome-wide chemical mutagenesis allied to next-generation sequencing. We show that chemically mutagenizing the genome of cancer cells dramatically increases the number of drug-resistant clones and allows the detection of both known and novel drug resistance mutations. We used an efficient computational process that allows for the rapid identification of involved pathways and druggable targets. Such a priori knowledge would greatly empower serial monitoring strategies for drug resistance in the clinic as well as the development of trials for drug-resistant patients.

Despite an increasing array of new cancer therapies, drug resistance is an almost universal phenomenon that is likely due to the presence of rare subclonal populations that act as a reservoir for resistance mutations. The emergence of drug resistance ultimately proves fatal for the majority of patients, and therefore the early detection of resistance and the identification of novel resensitization strategies is a subject of intense activity.

Previously, the identification of drug resistance genes has relied on either rebiopsy of cancer patients following the development of resistance or the use of cancer cell lines made resistant by exposure in vitro to drug over many weeks. Both approaches can suffer from inherent biases. With respect to the former, biopsy of a single resistant site of disease may miss alternate resistance mechanisms in other metastatic sites (Van Allen et al. 2014). Equally, serial drug exposure in cancer cell lines will favor preexisting drug-resistant clones that are specific for that cell line and may not represent the entire spectrum of resistance mechanisms for that treatment.

For these reasons, there is considerable interest in the use of forward genetic screens capable of engineering into the cancer genome mutational events that can be tested for their ability to cause drug resistance in an unbiased fashion. Such screens, if sufficiently unbiased, could in theory capture the entire breadth of genetic resistance mechanisms for any drug. Recent studies have demonstrated the power of both genome-wide gain- and loss-of-function screens using CRISPR/Cas9, lentiviral shRNA, and large-scale open-reading frame technologies to identify clinically relevant drug resistance mechanisms in cancer (Hu and Zhang 2016). However, these screens all fail to capture a third important mechanism of drug resistance, namely that of point mutations. Point mutations account for resistance in large numbers of patients receiving targeted therapies in melanoma, colon and lung cancers, and chronic myeloid leukemia (Supplemental Table S1; Kobayashi et al. 2005; Katayama et al. 2012; Montagut et al. 2012; Ohashi et al. 2012; Bettegowda 2014; Long et al. 2014; Van Allen et al. 2014; Wagle et al. 2014; Arena et al. 2015; Russo et al. 2015; Siravegna et al. 2015; Thress et al. 2015).

*N*-Ethyl-*N*-nitrosourea (ENU) has been used as a potent mutagen in mouse models of development for over four decades (Acevedo-Arozena et al. 2008). Exposure results in the efficient generation of random point mutations throughout the cell genome (Tokunaga et al. 2014). We therefore tested whether, in cancer cell line models, ENU could be used to mutagenize the genome and enable expansion of drug-resistant cells following the application of a targeted agent. As proof of concept, we chose to investigate whether this approach could identify all clinically demonstrated resistance mutations in colorectal cancer patients treated with the EGFR monoclonal antibody Cetuximab (Van Cutsem et al. 2009). In the clinic, resistance in such patients is heavily driven by point mutations, and a decade of clinical studies has identified the vast majority of the resistance mutations. This “ground truth” should in theory allow us to define how well a saturation mutagenesis screen can identify clinically relevant resistance mutations.

We used a sequencing and informatics approach to detect novel resistance mutations from next-generation sequence data and to detect statistical enrichment for mutually exclusive mutations in specific signaling pathways comprising more than 8000 genes at the sample population level. Our mutagenesis screen was able to successfully identify all known drug resistance mutations to Cetuximab previously observed in the clinic as well as a novel mutation that we subsequently identified in a colorectal cancer patient. We suggest that this approach is a powerful and facile means to draw the landscape of point mutations that confer resistance to targeted therapies. Such knowledge could be used to discover therapeutic strategies to resensitize resistant tumors as well as identify which genes should be prioritized for noninvasive monitoring during treatment using plasma DNA sequencing.

## Results

### ENU exposure confers stable resistance to Cetuximab in colon cancer cells

**Figure 1.**
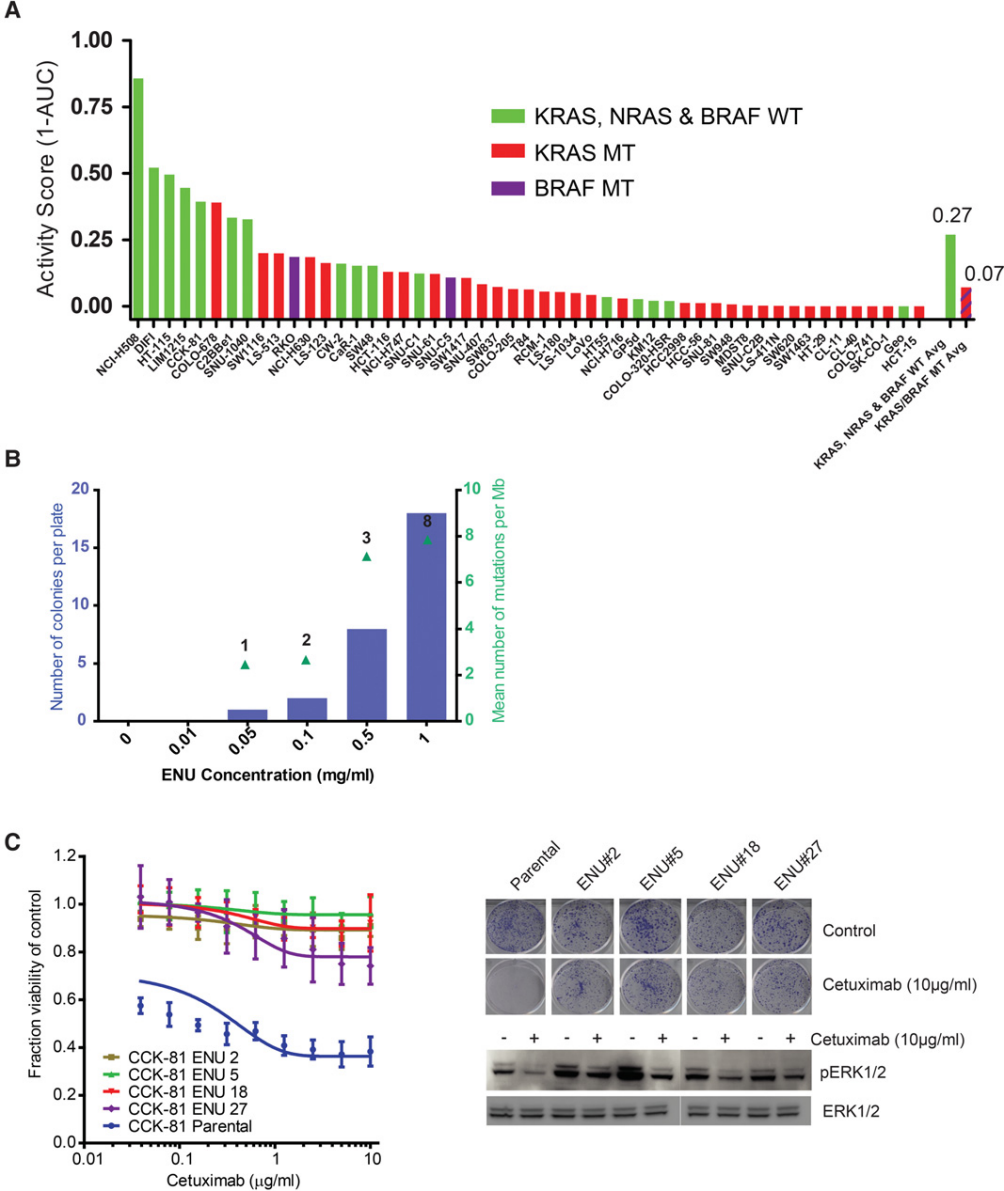
Genome-wide chemical mutagenesis screens to define pathways of drug resistance in cancer. (*A*) Cetuximab viability screen in colorectal cancer cell lines. Fifty-one colorectal cancer cell lines were screened with a concentration range of the EGFR monoclonal antibody Cetuximab and viability (as measured by the Activity Score, 1-AUC) was measured after 6 d. The *KRAS*, *NRAS*, and *BRAF* mutation status of each line is indicated. The mean Activity Score for those cell lines wild-type for all three genes (green bar) versus those with a canonical driver mutation in at least one gene (red bar) is indicated in the last two columns. (*B*) Dose-dependent effect of ENU on mutation burden and drug resistance. CCK-81 colorectal cancer cells were treated for 24 h with increasing concentrations of ENU (*x*-axis, 0.01–1 mg/mL) and then weekly with Cetuximab (10 µg/mL) for 8 wk to allow drug-resistant colonies to develop. The number of resistant colonies per plate were counted for each ENU concentration (blue bars) and submitted for whole-exome sequencing to calculate the mean number of mutations per Mb (green triangles). Numbers *above* each triangle indicate the number of clones sequenced at that concentration. (*C*) ENU mutagenesis generates stably drug-resistant clones. A subset of Cetuximab-resistant clones generated in the CCK-81 cell line following exposure to ENU and subsequent serial weekly Cetuximab treatment were picked from the plate and expanded separately out of drug for 4 wk. Twenty-one day clonogenic survival assays in four clones (along with the parental CCK-81 cell line) treated with Cetuximab 10 µg/mL confirmed that resistance to the EGFR monoclonal antibody had been maintained and was stable. A 6-d viability assay of cells treated with a concentration range of Cetuximab (*right*) demonstrates resistance of ENU clones at all concentrations. Immunoblot analysis of effect of Cetuximab treatment (10 µg/mL) for 6 h confirms persistence of MAPK signaling in ENU clones.

We screened 51 colorectal cancer cell lines with a concentration range of the EGFR monoclonal antibody Cetuximab and assessed viability after 6 d (Fig. 1A). In keeping with clinical experience of the genetic factors that underpin response to this drug, those cell lines wild-type for *KRAS/NRAS/BRAF* (green bars) exhibited heightened sensitivity to Cetuximab (Douillard et al. 2013). We therefore chose two of these lines, CCK-81 and NCI-H508, to use in the ENU resistance experiment. Both cell lines additionally demonstrated Cetuximab sensitivity in long-term clonogenic survival assays (Supplemental Fig. S1). Moreover, CCK-81 has features of microsatellite instability (MSI), whereas NCI-H508 is microsatellite stable (MSS). MSI is detected in 16% of colorectal cancers and is associated with a different phenotype and clinical outcome compared to MSS cancers. The CCK-81 cell line was exposed to a dose range of ENU (0.01–1 mg/mL) for 24 h, following which the mutagenized cells were treated with Cetuximab (10 µg/mL) for 8 consecutive weeks. The number of drug-resistant colonies was counted at the end of the experiment. Importantly, we observed no drug-resistant colonies in the absence of ENU (Fig. 1B). With increasing ENU concentration, we observed a linear increase in both the number of drug-resistant colonies (left *y*-axis, blue bars) as well as the number of mutations per clone (right *y* axis, green triangles). We subsequently used a concentration of ENU (0.1 mg/mL) that resulted in minimal viability effect in both cell lines (Methods). We next treated NCI-H508 cells with ENU (0.1 mg/mL) for 24 h followed by weekly Cetuximab treatment for eight weeks. Drug-resistant colonies were picked, expanded in culture, and 72 were submitted for whole-exome Illumina sequencing (a total of 14 CCK-81 and 58 NCI-H508 colonies). Data were analyzed for substitutions and insertions/deletions to enable an estimation of the number of ENU-associated mutations per Mb of exome and to detect novel (and putative drug resistance) mutations (Supplemental Table S2). We then performed clonogenic survival assays on a subset of resistant clones and confirmed robust and stable resistance to Cetuximab (Fig. 1C).

### The spectrum of ENU-induced mutations

**Figure 2.**
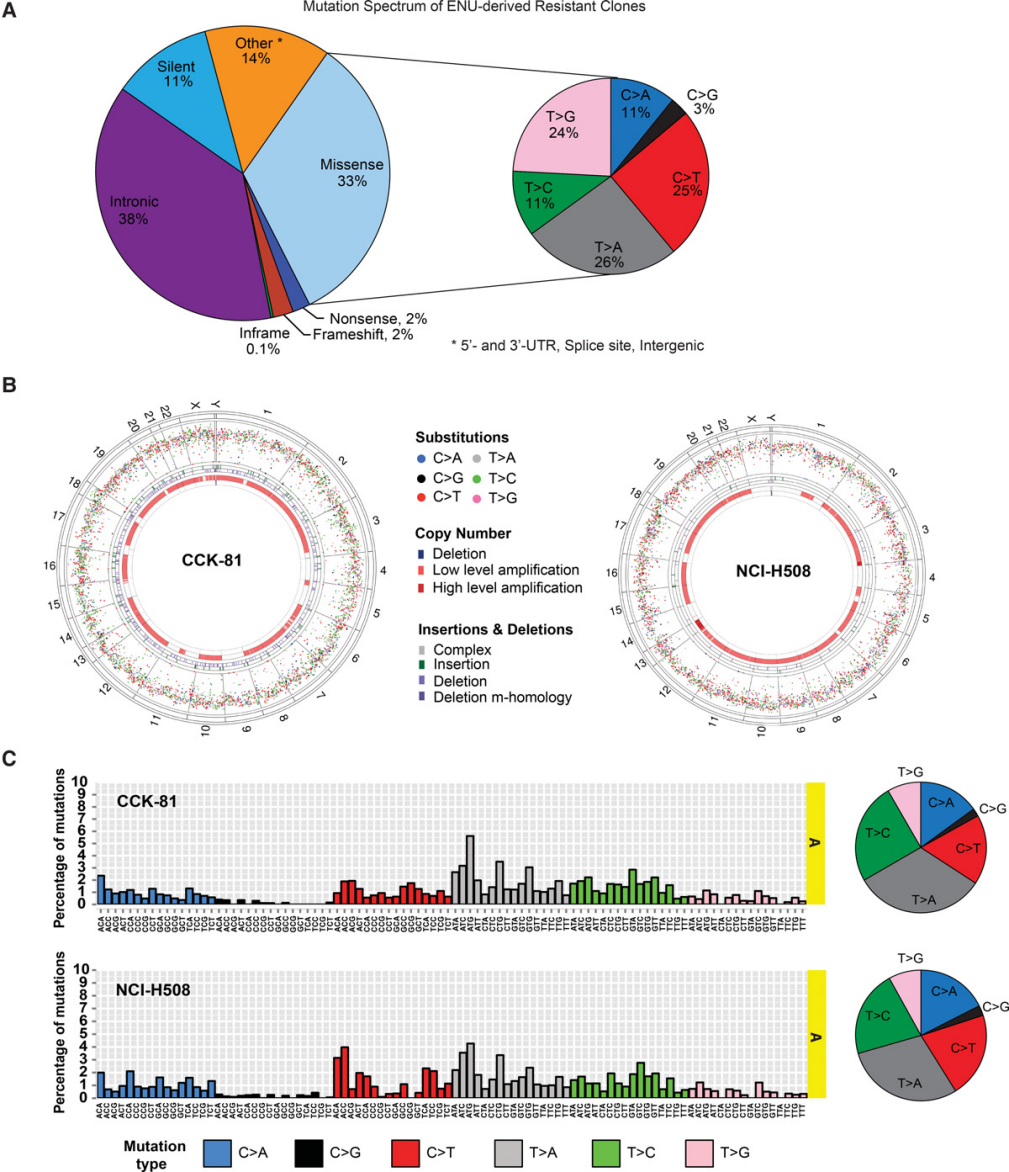
The spectrum of ENU-induced mutations. (*A*) Spectrum of mutations in ENU-derived drug-resistant clones. A pie chart representation of the proportion of 33,857 mutations detected in CCK-81 and NCI-H508 clones categorized according to mutation type. For the missense mutations, there is a further representation of the proportion of mutations falling within the six possible nucleotide base substitutions (C > A, C > G, C > T, T > A, T > C, T > G). (*B*) Circos plots of mutations in CCK-81 and NCI-H508 clones. All substitution mutations in CCK-81 or NCI-H508 clones are represented by their intermutation distance per chromosome. Each chromosome is composed of all coding exons in genes found within that chromosome. The four circles in each plot, from outermost inward, represent the human chromosomes, substitution mutations, insertion mutations, deletion mutations, copy number gains, and copy number losses. Low- and high-level amplification refers to 2–4 and 4–8 copies, respectively. (*C*) The mutational signature of ENU. The trinucleotide representation of ENU mutations in CCK-81 and NCI-H508 drug-resistant colonies is displayed as the distribution of mutations for all 96 possible combinations of mutations generated as a result of ENU exposure. Adjacent pie charts display the relative distribution of each of the six classes of possible base substitutions for the CCK-81 and NCI-H508 clones.

The ability of any mutagenesis screen to capture a particular phenotype is strongly dependent on its ability to evenly saturate the genome with all six possible classes of base substitution type (expressed as the pyrimidine of a mutated Watson-Crick base pair, C > A, C > G, C > T, T > A, T > C, T > G). On average, we detected 470 novel mutations per exome in each clone (mean 570 and 446 in CCK-81 and NCI-H508 clones, respectively), for a total of 33,857 (Supplemental Table S2). The mutations were almost exclusively composed of base substitutions (96% of total). A third of such mutations were nonsynonymous (missense) variants within the coding exon of a gene, where resistance mutations are more likely to occur (Fig. 2A). Only 4% were potential loss-of-function truncating mutations (frameshift indels or nonsense mutations). The remaining mutations were predominantly either silent or intronic. Significantly, analysis of exome sequence data across all 72 clones (regardless of whether MSS or MSI) revealed that of the six possible classes of base substitution, only C > G substitutions are less well represented (3% of all substitution base changes) (Fig. 2A). The mutation spectrum in ENU-derived clones was similar regardless of whether the cells came from a MSI or MSS background (Supplemental Fig. S2). There was no evidence of a significant bias toward mutations in coding genes in any particular chromosome or indeed any specific region within a chromosome (Fig. 2B; Supplemental Fig. S3). However, given these 33,857 mutations are an admixture of those caused by ENU, background mutational processes, and private subclonal variants, we elected to use a mathematical approach to specifically extract the ENU signature from the data and more accurately determine the mutation spectrum of ENU mutations.

The non-negative matrix factorization algorithm has previously been used to detect the presence of mutational signatures in human cancers, including from defects in DNA mismatch repair and altered activity of the error-prone polymerase *POLE* (http://cancer.sanger.ac.uk/cosmic/signatures) (Alexandrov et al. 2013b). It extracts signatures based on a 96-mutation classification that incorporates the six base substitution types described above as well as the immediate flanking sequence context of the mutated base (four possible 5′ and four possible 3′ bases). In our data, it revealed a distinct and unique signature that was represented across almost all trinucleotide contexts in both CCK-81 and NCI-H508 clones and not previously detected in prior tumor studies, including a panel of 51 colorectal cancer cell lines (data not shown) (Fig. 2C; Supplemental Fig. S4). This signature (“Signature A”) is likely one of ENU exposure. Reassuringly, the pattern of base substitutions that comprise this signature was almost identical to that seen across the entire set of substitutions detected in the ENU-derived clones, with again only C > G substitutions seen at lower frequency (Fig. 2C). Thus, using this approach it should be feasible to generate the majority of theoretical coding point mutations for drug resistance across the entire genome.

As expected, we detected a signature of MSI (“Signature B”) in the combined mutational catalog for the CCK-81 clones but surprisingly also in the NCI-H508 clones (Supplemental Fig. S4; Alexandrov et al. 2013a). On closer examination, this signature was the result of two hypermutator clones in the NCI-H508 mutational catalog (red arrows) (clones NCI-H508_26 and NCI-H508_40) (Supplemental Fig. S5). These clones had mutation rates as high as any of the MSI CCK-81 clones and increased numbers of small insertions and deletions. This would be in keeping with a defect in the mismatch repair pathway (Supplemental Table S2). Clone NCI-H508_26 was found to harbor a novel nonsense (stop-gained) mutation in the mismatch repair gene *MLH1*, and clone NCI-H508_40 harbored a nonsense mutation in the DNA repair gene *EXO1*. Two other clones (black arrows) also have elevated mutation rates that may be the result of gaining nonsense mutations in *POLQ*, a gene involved in DNA damage repair. These gave rise to the third signature of unknown origin detected in NCI-H508 clones (“Signature C”) (Supplemental Fig. S4).

### ENU mutagenesis identifies clinically relevant resistance mutations and pathways

**Figure 3.**
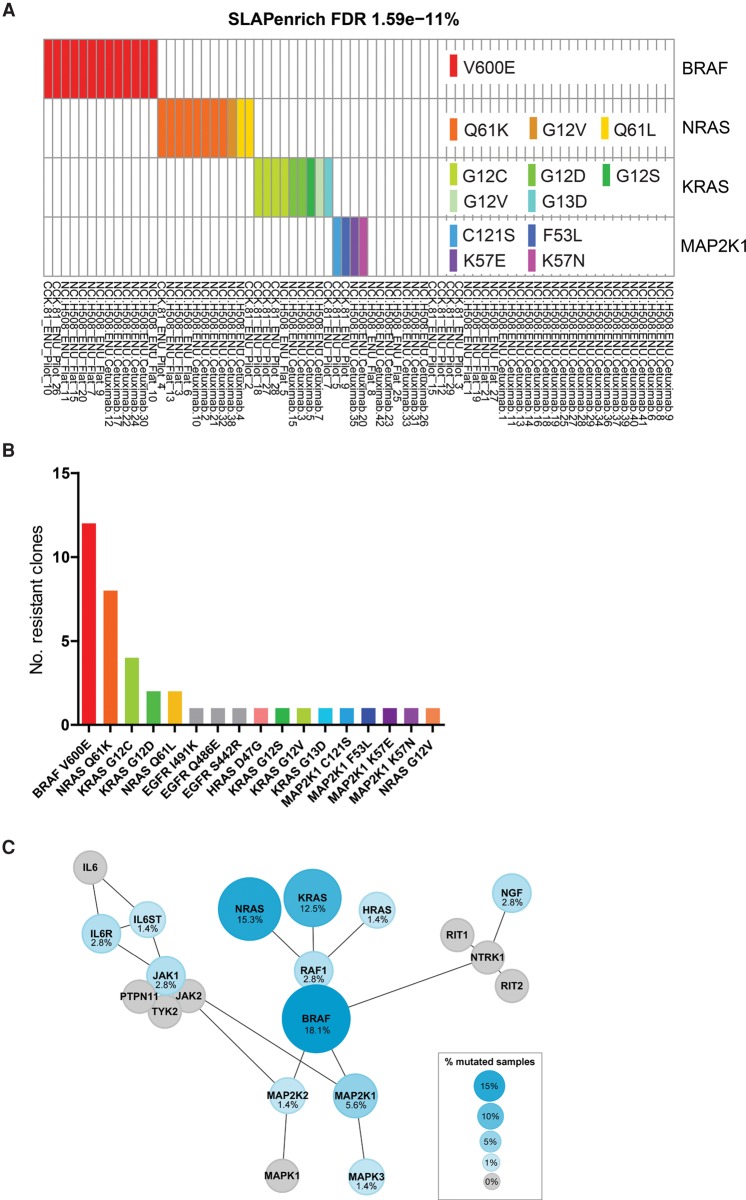
ENU mutagenesis identifies clinically relevant mutations and pathways. (*A*) The pathway most significantly enriched with mutations, “Signaling to P38 via RIT and RIN,” contains many of the key genes of the canonical MAP kinase pathway, and those are mutated in a mutually exclusive manner. Here, we show those genes mutated in at least three individual clones. Whole-exome sequence from 72 Cetuximab resistant ENU clones was used to identify pathways using the SLAPenrich algorithm. Amino acid substitutions are labeled for the subset of genes most frequently mutated in the pathway and/or demonstrating mutual exclusivity with other mutations. (*B*) Cetuximab resistance mutations. Frequency of likely ENU-derived drug resistance mutations across 42 resistant clones. The amino acid consequence of each mutation is indicated with the gene name. (*C*) A visual representation of the all mutated genes that comprise the “Signaling to P38 via RIT and RIN” pathway, indicating gene–gene interactions and the hierarchy of signaling. Only those genes mutated in at least three clones were taken forward for validation.

A challenge in the identification of drug resistance mutations in ENU-derived clones is that each clone harbors many hundreds of “passenger” mutations in addition to that conferring resistance. We hypothesized that with a sufficient population of individual resistant clones it might become feasible to use statistical enrichment for nonsynonymous coding mutations in specific pathways to help identify drug resistance mutations. We therefore used a statistical framework (SLAPenrich) to identify whether genetic alterations observed in multiple samples are enriched within a specific pathway in a statistically significant manner using a network of 8056 unique genes (https://github.com/saezlab/SLAPenrich) (Iorio et al. 2016). Once significantly enriched pathways are identified, SLAPenrich applies a final filter based on the tendency for genes in a positively selected pathway to be mutated in a mutually exclusive manner. When applying this method to the set of ENU-mutations across the 72 Cetuximab-resistant clones, we found several statistically enriched pathways (false discovery rate [FDR] < 5%) (Supplemental Table S3). The pathway most significantly enriched with mutations, “Signaling to P38 via RIT and RIN,” contains many of the key genes of the canonical MAP kinase pathway (Fig. 3A). In total, we were able to identify credible resistance mutations in 42 of the 72 resistant clones (59%) (Supplemental Table S4). We detected credible resistance mutations in all of the genes previously found clinically to confer resistance to EGFR therapy in colorectal cancer (Supplemental Table S1). *EGFR, KRAS, NRAS, BRAF*, and *MAP2K1* (also known as *MEK1*) were each found to be mutated in three or more clones and in a mutually exclusive manner. There was no clear difference in the frequency of specific mutations between NCI-H508 and CCK-81. Furthermore, 38/42 (90%) of these putative resistance mutations have previously been identified in colorectal patients developing resistance to Cetuximab. The most frequently observed ENU resistance mutation was that of *BRAF* p.V600E (13/42 clones), followed by *NRAS* p.Q61K (8/42) and *KRAS* p.G12C (4/42) (Fig. 3B,C; Supplemental Fig. S6). These mutations are all canonical driver mutations in tumorigenesis and known to activate oncogenic signaling and confer resistance to Cetuximab both experimentally and clinically (Diaz et al. 2012; Misale et al. 2012). We also detected *EGFR* mutations in three of the resistant clones. The *EGFR* I491K substitution has been shown to induce structural changes to the extracellular domain of EGFR such as to prevent Cetuximab binding and confer resistance (Montagut et al. 2012). Of note, additional mutations also present in the “Signaling to P38 via RIT and RIN” network (e.g., JAK1, IL6R, RAF1) were not taken forward for investigation as these mutations were present in clones also harboring other more credible drivers of drug resistance and were present at low frequency (fewer than three clones) (Supplemental Fig. S7).

### A mutation enrichment analysis identifies drug resistance genes

Recent studies of large mutational data sets from cancer sequencing studies have used statistical approaches that consider the mutation spectrum, the sequence of each gene, the impact of coding substitutions (synonymous, missense, nonsense, splice site), and the variation of the mutation rate to detect novel cancer genes (Nik-Zainal et al. 2016). We adapted this “*d*_N_/*d*_S_” (nonsynonymous/synonymous) method to analyze the mutations identified in the ENU drug-resistant clones (Methods). As this approach was designed for the analysis of unrelated tumor samples and we have instances of clones sharing mutations that reflect a common subclonal origin within the original parental cell lines, we first condensed the 72 ENU clones into 19 representative groups, each the union of all the mutations in clones sharing more than three mutations. Three genes were identified as having a pattern of mutations supporting being under positive selection, namely *NRAS, KRAS*, and *MAP2K1* (FDR < 0.05) (Supplemental Table S5). After these, the next highest ranked gene was *BRAF*; although it was of borderline significance (FDR = 0.0508), it is a very strong candidate for a resistance gene because 4/6 of the mutations are at the canonical p.V600 locus.

### ENU mutagenesis identifies novel resistance mutations in *MAP2K1*

**Figure 4.**
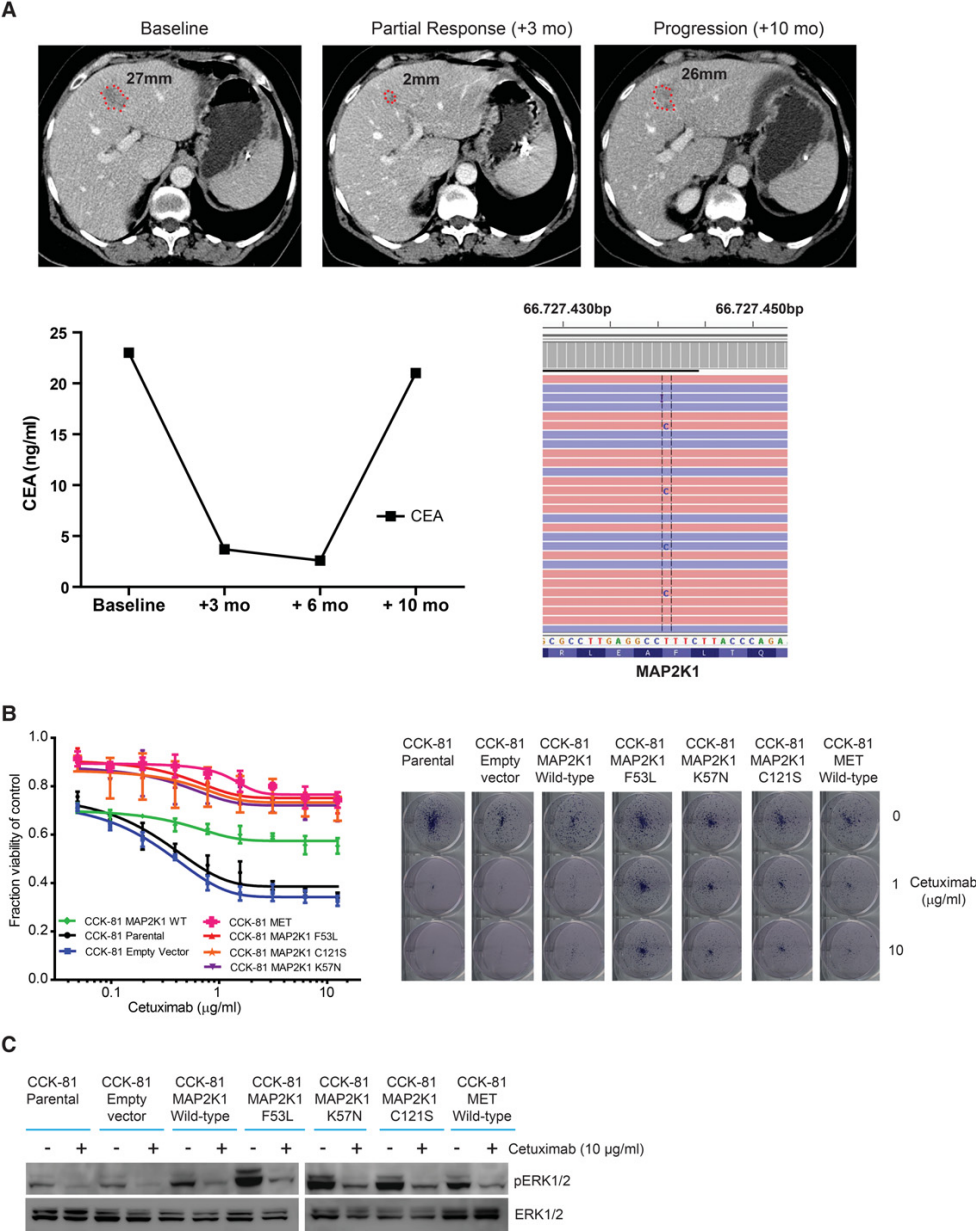
ENU mutagenesis identifies a novel *MAP2K1* resistance mutation that is detected in a colorectal cancer patient after an initial response to Cetuximab. (*A*) A colorectal cancer patient with inoperable liver metastases was treated with the combination of FOLFIRI chemotherapy and Cetuximab. Initial response to treatment was demonstrated radiologically at 3 mo and by falling CEA marker levels. Subsequent measurements at 10 mo confirmed progressive disease, and sequencing of plasma DNA detected a novel mutation in *MAP2K1*, p.F53L. (*B*) CCK-81 cells were transduced with the following lentiviral *MAP2K1* vectors: empty vector (mock), wild-type (WT), K57N, C121S, and F53L mutations. MET was also overexpressed as a positive control. Cells were seeded in 96-well plates and treated for 1 wk with increasing concentrations of Cetuximab (0–12.5 µg/mL) (*left*) or assayed using a clonogenic survival assay at 20 d at 1 or 10 µg/mL of Cetuximab (*right*). (*C*) Immunoblot of the effect of Cetuximab (10 µg/mL) at 6 h on pERK in *MAP2K1* mutant CCK-81 cells (as in *B*).

Recently, two studies of plasma DNA sequencing in colorectal cancer patients undergoing treatment with EGFR monoclonal antibodies jointly identified the first *MAP2K1* codon K57 resistance mutations (p.K57T and p.K57N) (Russo et al. 2015; Siravegna et al. 2015). In our study, we also identified *MAP2K1* mutations at the K57 codon (p.K57N, p.K57E) as well as at two sites not previously reported (p.F53L, p.C121S) (Supplemental Table S4). We therefore sequenced these *MAP2K1* loci (together with additional mutation hotspots in 34 other genes) in a series of plasma DNA samples collected from 22 colorectal cancer patients who acquired resistance to treatment with EGFR therapies (either Cetuximab or Panitumumab) after an initial response. In addition to all of the known canonical resistance mutations (in *KRAS, NRAS*, and *BRAF*), we detected in one such patient a novel p.F53L *MAP2K1* mutation predicted by our screen to be a resistance mutation (Table 1; Fig. 4A). As previously reported, we detected more than one likely resistance mutation in a number of these patients, in keeping with different metastatic sites evolving different resistance mechanisms.

**Table 1.**
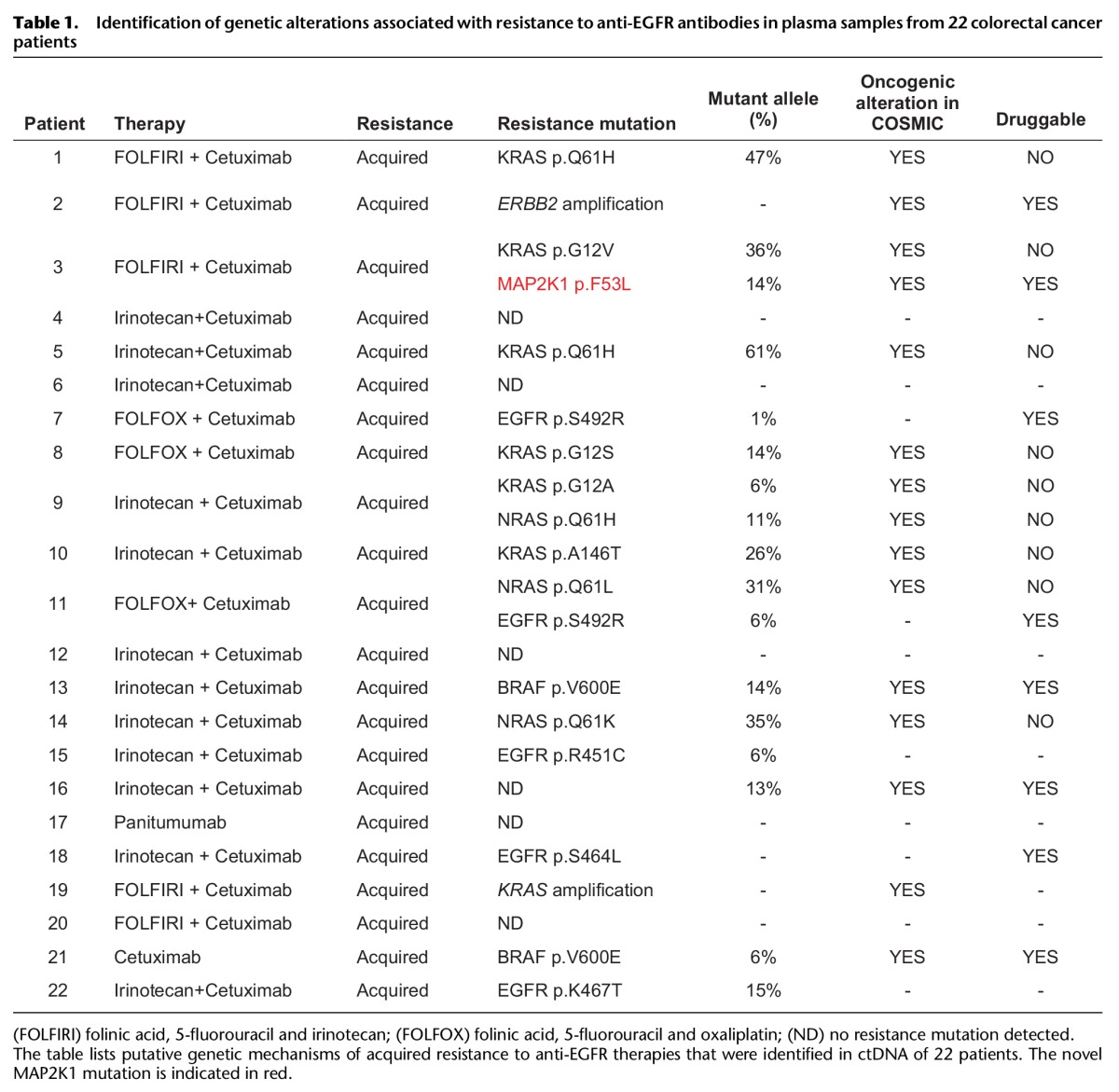
Identification of genetic alterations associated with resistance to anti-EGFR antibodies in plasma samples from 22 colorectal cancer patients

To functionally validate the resistance effects of these *MAP2K1* mutations, we treated CCK-81 cells expressing the novel p.F53L and p.C121S mutations as well as the previously identified p.K57N mutation with Cetuximab (alongside empty vector and wild-type *MAP2K1* controls). We found that all of our candidate resistance mutations induced resistance to Cetuximab, and the strength of the resistance effect for the mutations was comparable to that conferred by overexpression of the MET receptor tyrosine kinase, a previously identified resistance mechanism (Fig. 4B, left; Bardelli et al. 2013). Long-term growth inhibition assays similarly showed robust and durable resistance to Cetuximab in the *MAP2K1* mutant cells (Fig. 4B, right). Immunoblot analysis demonstrated elevated constitutive phosphorylation of ERK1/2 as well as a failure to completely suppress pERK1/2 expression following Cetuximab treatment in all of the *MAP2K1* mutant clones (Fig. 4C).

### Rational targeting of pathways can resensitize drug-resistant mutants to Cetuximab

**Figure 5.**
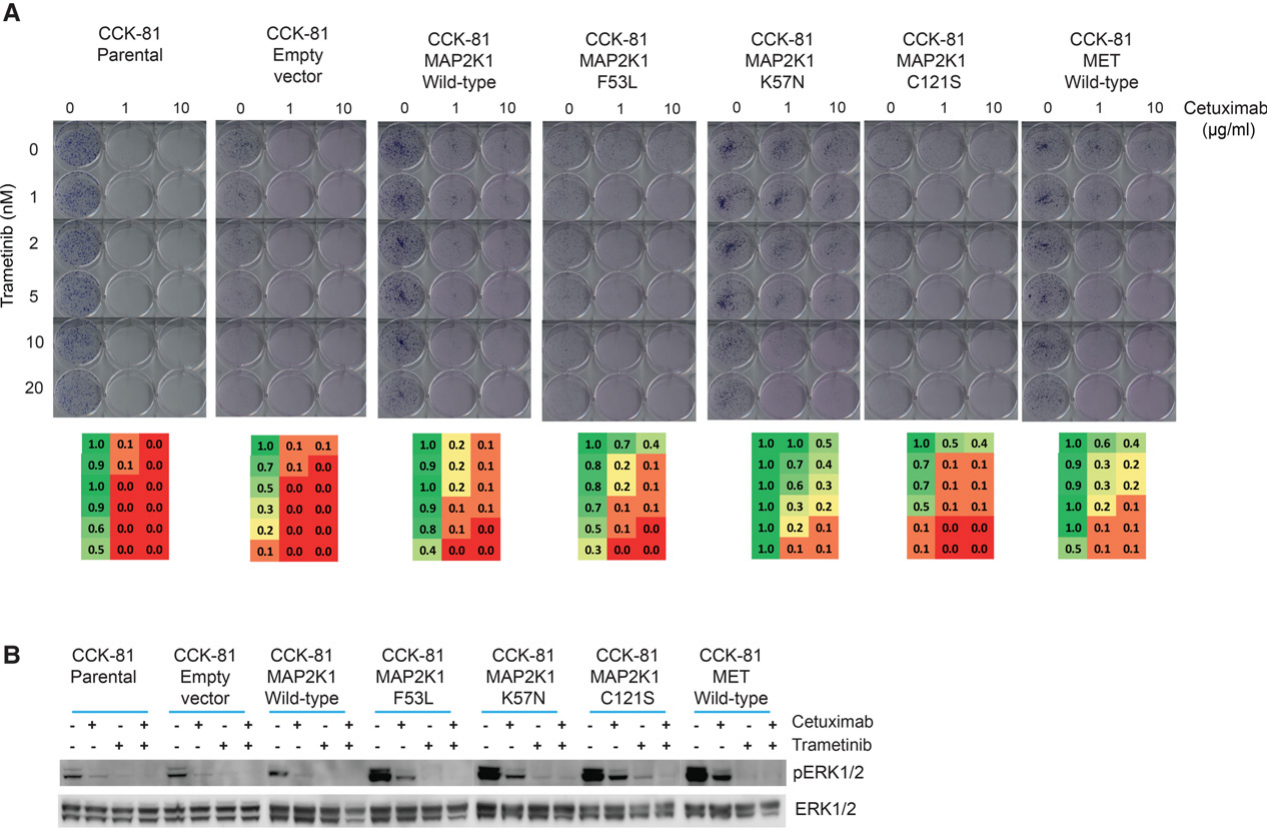
Rational targeting of resistance pathways to resensitize drug-resistant mutants. (*A*) Clonogenic survival assay of *MAP2K1* mutant CCK-81 cells when treated with Cetuximab, Trametinib, or a combination of the two drugs. Heat maps of normalized viability effects per well are shown *below* each cell line. (*B*) Immunoblot of the effect of combing Cetuximab and Trametinib (MEK inhibitor) on MAPK signaling in *MAP2K1* mutant CCK-81 cells. Cells were treated with Cetuximab (10 µg/mL), Trametinib (5 nM), or both for 6 h. MET was also overexpressed as a positive control.

Constitutive EGFR signaling in solid tumors activates a number of downstream pro-survival/proliferation pathways including PKC, PI3K/AKT/mTOR, JAK-STAT, and MAPK (Supplemental Fig. S8A; Shostak and Chariot 2015). In EGFR-dependent cells, treatment with EGFR inhibitors affects cell survival by shutting down such processes. Identification of the key signaling pathways that underpin drug resistance opens up the possibility of rationally targeting key components of such resistance pathways and thus resensitizing cells. The creation of mutagenized resistant cell lines, either through the ENU screen or through the deliberate genetic modification of the parental cell line for specific mutations, allowed us the opportunity for such experiments to be carried out in vitro. As in clinical practice, the pathway most frequently mutated in the drug-resistant CCK-81 and NCI-H508 ENU clones converges toward MAPK family members, and targeting these nodes might be expected to overcome resistance (Fig. 3C). For example, a Cetuximab-resistant CCK-81 *BRAF* V600E mutant clone (ENU-10) was resensitized when the EGFR monoclonal antibody was combined with the BRAF inhibitor Dabrafenib (Supplemental Fig. S9A). In the mutant cells, the activating *BRAF* mutation would enable such cells to continue to signal through the MAPK pathway (and survive) despite EGFR blockade (Supplemental Fig. S8B). Thus, only by targeting both EGFR and BRAF in combination can all the relevant survival effectors be silenced and the viability of the cells reduced. Cetuximab-resistant clones harboring mutations (in *KRAS, NRAS*, and *MAP2K1*) that would be predicted to activate MAPK signaling were resensitized when a MEK inhibitor (Trametinib) was combined with Cetuximab (Supplemental Fig. S9B). Similarly, combining Cetuximab with Trametinib almost completely resensitized the resistant *MAP2K1* mutant CCK-81 cells (Fig. 5A,B). Indeed, such a combination has already been suggested as putative therapeutic strategy for colon cancer patients (Misale et al. 2014).

## Discussion

We proposed at the onset of this study that regardless of what sequencing technology is used to detect resistance point mutations, some a priori knowledge of the likely drug resistance candidates should greatly increase the sensitivity of such assays. Identifying the complete catalog of drug resistance effectors to any drug requires in vitro studies that model resistance in the relevant tissue and genetic background. In the past, such in vitro studies featured cell lines that had undergone serial passage in the presence of the candidate drug in order to force the emergence of resistant clones (Ogino et al. 2007; Turke et al. 2010; Katayama et al. 2011; Eberlein et al. 2015). Although such studies have successfully identified clinically relevant mechanisms of drug resistance in some instances, they are biased toward selecting for those preexisting resistant subclones that are particular to that specific cell line.

As a means of generating random point mutations throughout the genome, ENU chemical mutagenesis and subsequent phenotype-driven screening has been pivotal to a complete understanding of how complex biological processes operate in classical model organisms including yeast (Forsburg 2001), flies (St Johnston 2002), zebrafish (Patton and Zon 2001), and, perhaps most extensively, mice (Kile and Hilton 2005). The alkylating agent *N*-ethyl-*N*-nitrosourea (ENU) can introduce a high rate of point mutations into the genome and has two distinct advantages over previously used mutagens. First, it is very efficient, inducing a point mutation every 1–2 Mb throughout the genome in mouse models (∼100-fold higher than the spontaneous mutation rate and threefold higher than X-irradiation) (Concepcion et al. 2004). Second, unlike irradiation, which induces multilocus deletions, ENU is a point mutagen and affects single loci. ENU functions by the transfer of its ethyl group to oxygen or nitrogen atoms in DNA, resulting in misidentification of these ethylated bases during replication. If the mismatch is not repaired, a base-pair substitution results (Justice 2000). To date, the use of ENU to define drug resistance mechanisms in cancer has been focused on specific genes in noncancer cell line models rather than to interrogate the entire coding genome (Tiedt et al. 2011; Zhang et al. 2011; Ercan et al. 2015). Previous sequencing studies of ENU-derived mutations in mouse and fly models demonstrated a strong bias in the spectrum of substitution mutations observed, with especially low numbers of C > G, T > G and C > A mutations. We observed a much more balanced representation of all six possible base substitution contexts in our human cancer cells following ENU exposure (Supplemental Table S8). There should therefore be greater potential to identify a larger number of resistance mutations regardless of their mutation spectrum. Additionally, we compared the mutation spectrum of ENU to that of another mutagen, namely gamma irradiation. Two cancer cell lines (MSI versus MSS) were irradiated, and single cells were expanded to clones prior to exome sequencing. We observed that the mutation spectrum differed quite significantly for the two cell lines, suggesting that unlike ENU mutagenesis, there is a greater cell line–specific effect on the pattern of mutations observed (Supplemental Fig. S10). This would need to be taken into account if gamma irradiation was used for detection of drug resistance genes.

Because it is likely that only one allele is mutated for any specific gene, and <5% of the ENU mutations are capable of abrogating protein expression (i.e., nonsense, frameshift, or essential splice site mutations), the screen is strongly biased toward gain-of-function or dominant point mutations. Loss-of-function resistance genes would therefore be better captured through genome-wide CRISPR inactivation screens or to use ENU in the setting of haploid cell models (although how relevant these are for aneuploidy cancer cells might be a confounding issue).

A major challenge in the interpretation phenotype-directed screening of ENU mutation models is to identify driver mutations from passenger mutations. This is of particular importance in our experiment because ENU mutagenesis generates an average of almost 500 new mutations per drug-resistant clone. We posited that in addition to recurrence, evidence that multiple mutations were enriched within the same network or pathway in a mutually exclusive manner would increase the likelihood of these being driver events. There is a long history of using public resources of such networks to identify enriched genes. We used an algorithm (SLAPenrich) to model the likelihood of observing a given number of samples with mutations in the pathway under consideration through a Poisson binomial distribution. It is important to note that identifying resistance mutations in such ENU mutagenesis data is heavily dependent on recurrence of the same mutations across multiple samples; therefore, identification of rare resistance mutations will be challenging unless massive numbers of resistance clones are generated and sequenced. This is especially pertinent with respect to the 30 drug-resistant CCK-1 or NCH-H508 clones, in which SLAPenrich was unable to detect statistical enrichment of mutations in specific pathways. We suggest at least two plausible explanations: (1) These are rare (and therefore not recurrent) mutations and therefore do not cluster into previously characterized pathways interrogated by SLAPenrich; and (2) the observed resistance is the result of mutations outside of the coding exome (for example, in enhancer/promoter or untranslated [UTR] regions) and therefore not amenable to detection using this whole-exome capture approach. With respect to the former possibility, we re-ran SLAPenrich after removing any variants in the previously identified putative resistance genes (*BRAF, KRAS, NRAS, MAP2K1*, etc.) to increase the power to detect additional enriched pathways; we found nine pathways that were enriched when considering a far less stringent significance threshold (FDR < 20%). These were dominated by pathways of neurotransmitter signaling (Supplemental Table S9). Given what we know today about EGFR signaling, these are not plausible candidates to confer resistance to Cetuximab. Finally, investigating the possibility of noncoding mutations as resistance drivers would require whole-genome sequencing of these hypermutated samples (each harboring ∼50,000 mutations per genome) and the development of statistical approaches for recurrence detection in noncoding regions. Such algorithms are currently under development as part of the PanCancer Analysis of Whole Genomes (http://pancancer.info/) which is undertaking the analysis of more than 2000 whole genomes. Thus, in due course as whole-genome sequencing costs fall and analytical tools are developed for the noncoding genome, these ENU clones that harbor possible noncoding resistance point mutations could be resequenced and reanalyzed.

Here, we establish a model for the use of genome-wide chemical mutagenesis screens to capture the diversity of clinically relevant drug resistance protein-coding mutations in aneuploid cancer cells. As proof of concept, we used this screen in the setting of an EGFR therapy and colorectal cancer, a disease in which response to such therapy is invariably followed by the acquisition of resistance. Such resistance mechanisms are heavily dominated by point mutations in the MAP kinase signaling pathway and have been extensively validated in patient cohorts (Supplemental Table S1; Yonesaka et al. 2011; Diaz et al. 2012; Misale et al. 2012; Montagut et al. 2012; Bardelli et al. 2013; Bettegowda 2014). We are able to identify all clinically detected resistance mutations to Cetuximab treatment in colorectal cancer, and in addition, potential therapeutic avenues to resensitize resistant cells. We propose that ENU mutagenesis should be incorporated alongside newer genome-wide CRISPR gene editing technologies in the systematic interrogation of drug resistance given the prevalence (and potential for therapeutic targeting) of point mutations as mediators of resistance in cancer.

## Methods

### Materials

All cell culture was performed in either RPMI or DMEM/F12 medium (according to the supplier's recommendations) and supplemented with 5% FBS and penicillin/streptavidin. Cells were maintained at 37°C and 5% CO_2_ during culture. The identity of all cell lines used in this paper was confirmed using a panel of 95 single nucleotide polymorphisms (SNPs) used previously for cell line authentication (Fluidigm).

### Immunoblotting

Differential phosphorylation of proteins in signaling pathways were analyzed by Western blot. Cells were plated 24 h prior to drug treatment and incubated for indicated times and concentrations. Adherent cells were then washed with PBS and collected after indicated incubation time with drug using lysis buffer containing 5% β-mercaptoethanol, 150 mM NaCl, 50 nM Tris pH 7.5, 2 nM EDTA pH 8, 25 nM NaF, 1% NP-40, protease inhibitors (Roche), and phosphatase inhibitors (Roche). Lysates were then normalized after bicinchoninic acid (BCA) assay using lysis buffer. Protein lysates were resolved using SDS PAGE electrophoresis in precast Invitrogen 4%–12% Bis-Tris gels and transferred for 12 h. Primary antibodies: p44/42 MAPK, Phospho-p44/42 MAPK (Thr202/Tyr204), and AKT were sourced from Cell Signaling, and phospho-AKT (pS473) was sourced from Invitrogen. Monoclonal beta tubulin was sourced from Sigma.

### Drug sensitivity assays

Cells were seeded in 96-well plates for 6-d assays and six-well plates for 20-d clonogenic assays. Cells were incubated in drug-free media to allow for adherence for 24 h before the addition of drug at indicated concentrations. Each cell line was seeded to achieve ∼70% confluency at the end of the assay. Cetuximab was obtained from the Addenbrookes’ Hospital Pharmacy. Trametinib (GSK1120212) and Dabrafenib were obtained from Selleckchem.

### ENU mutagenesis of cell lines

The CCK-81 and NCI-H508 cell lines were incubated in a concentration range of ENU (0–10 mg/mL), and viability was measured after 48 h. A concentration of 0.1 mg/mL was subsequently selected for resistance models as having a modest effect on cell viability while still generating a high rate of mutations (Supplemental Fig. S11). Cells were incubated in ENU at the indicated concentration for 24 h before being washed three times with PBS and incubated in media for a further 24 h. Cells were then selected with 10 µg/mL Cetuximab 48 h post-ENU exposure for 8 wk. Clones were then picked using Scienceware small cloning cylinders and either transferred to 96-well plates or expanded into large flasks for drug sensitivity assays. DNA was extracted in 96-well plate format using the Agencourt DNAdvance Genomic DNA Isolation kit.

### Gamma radiation of cell lines

The MSI colon cancer cell line HCT116 and the MSS lung cancer line NCI-H3122 were each irradiated with either 1 Gy or 10 Gy. The following day, single cells were flow sorted and expanded as colonies. DNA was extracted from nine colonies from each cell line and submitted for sequencing.

### Whole-exome sequencing

Exome sequencing was carried out using the Agilent SureSelectXT Human All Exon 50-Mb bait set. Seventy-two clones were DNA extracted and underwent library construction, flow cell preparation, and cluster generation according to the Illumina library preparation protocol. We performed 75-base paired-end Illumina sequencing. Read alignment to the reference human genome (GRCh37) was performed using the Burrows–Wheeler Aligner (BWA) (http://bio-bwa.sourceforge.net/) (Li and Durbin 2010). Unmapped reads were excluded from the analysis. The average coverage across CCK-81- and NCI-508-derived clones was 65× and 62×, respectively. The matched parental cell lines were sequenced at greater depth (158× in CCK-81 and 144× in NCI-H508).

### Variant detection

Single-nucleotide substitutions were called using the CaVEMan C (Cancer Variants through Expectations Maximisation) algorithm, and insertions/deletions were called using split-read mapping implemented in the Pindel algorithm (https://github.com/cancerit). The CaVEMan algorithm only analyzes reads that are properly paired and not marked as duplicates. Variants were identified by comparison to a reference single-matched sample consisting of a high sequence coverage contemporary parental cell line control.

### Data filtering to remove preexisting subclonal variants

A number of clones shared mutations which were present in a small percentage of reads in their corresponding contemporary parental cell line sequence. These subclonal mutations could confound subsequent pathway analysis by causing enrichment in a pathway due to mutations that were present before ENU treatment but were not called due to their low representation. To overcome this problem, variants were filtered against the deep sequenced contemporary parental control after mutation calling via CaVEMan and Pindel. The SAMtools mpileup algorithm was used to remove any mutations which were present in 0.5% or more reads in the high coverage parental cell line control (Li et al. 2009). The final set of mutations was used to generate an event matrix for all 72 clones (Supplemental Table S6) and used as the input file for the SLAPenrich analysis described below.

### Deciphering mutational signatures branding exome sequences of clones exposed to ENU

The immediate 5′ and 3′ sequence context of base substitutions identified across Cetuximab-resistant clones was extracted using the Ensembl Core APIs for human genome build GRCh37 and was used to generate mutational catalogs for the downstream analysis. The mutational catalog of CCK-81 Cetuximab-resistant clones contained 7198 substitutions, whereas the NCI-H508 clones contained a total of 23,862 substitutions. Mutational signatures were deciphered separately across both catalogs of mutations, using a previously developed computational framework (Alexandrov et al. 2013b). Briefly, the algorithm identifies a minimal set of mutational signatures that optimally explains the proportions of mutation types found across a given mutational catalog (i.e., across all substitutions identified in CCK-81 and NCI-H508 clones) (Supplemental Fig. S4) and then estimates the contribution of each identified signature to a mutation spectra of each sample included in analysis (i.e., to a mutation spectra of each individual clone; see for NCI-H508 clones) (Supplemental Fig. S5).

### Sample level analysis of pathway enrichments (SLAPenrich)

A short description of the statistical model implemented in SLAPenrich is included in the Supplemental Methods, together with specifications of input and parameter settings used for the analyses presented in this manuscript. SLAPenrich is implemented as an R package, and it is publicly available at https://github.com/saezlab/SLAPenrich. The computational pipeline to reproduce the presented results is implemented in the enclosed BrammeldEtAl_analysis.R script (also available in the Supplemental Methods).

Briefly, as a first step, SLAPenrich estimates the probability of observing at least one gene belonging to a given pathway mutated in a given sample, based on the length of the total exon blocks of the genes in that pathway, and the sample mutation burden. Once this probability has been estimated for each individual sample, SLAPenrich models the likelihood of observing a given number of samples with mutations in the pathway under consideration through a Poisson binomial distribution. This is the discrete distribution of a sum of Bernoulli trials in which the probability of success is not constant. It is used by SLAPenrich to compute the deviance of the number of observed samples with mutations in a given pathway from its expectation through a corresponding *P*-value assignment.

### Identification of drug resistance genes based on the impact of coding mutations

To identify recurrently mutated driver genes, a *d*_N_/*d*_S_ method that considers the mutation spectrum, the sequence of each gene, the impact of coding substitutions (synonymous, missense, nonsense, splice site), and the variation of the mutation rate across genes were used. Owing to the lack of a neutral reference for the indel rate in coding sequences, a different approach was required (for details, see Supplemental Methods). Because this approach has been developed for the detection of driver genes in large cancer sample data sets, samples sharing more than three mutations (i.e., related subclonal populations) were merged to create a single set of variants composed of the union of all observed variants in the similar samples. In the case of the 72 ENU clones, this resulted in the creation of 19 informative samples that were used for the *d*_N_/*d*_S_ analysis.

To detect genes under significant selective pressure by either point mutations or indels, for each gene, the *P*-values from the *d*_N_/*d*_S_ analysis of substitutions and from the recurrence analysis of indels were combined using Fisher's method (Fisher 1922). Multiple testing correction (Benjamini-Hochberg FDR) was performed separately for all genes, stratifying the FDR correction to increase sensitivity (Benjamini and Hochberg 1995; Sun et al. 2006). To achieve a low false discovery rate, a conservative *Q*-value cutoff of <0.05 was used for significance and considered significant any gene with qmis_sfdr<0.05 OR qglobal_sfdr<0.05. Please see Supplemental Methods for detailed explanations of these methods.

### Site-directed mutagenesis of *MAP2K1* expression vectors

In order to validate candidate drug resistance mutations from the ENU-based forward genetic screen, we sought to create mutated vectors to express within Cetuximab-sensitive colorectal cell lines. Wild-type construct for *MAP2K1* was ordered from Dharmacon and taken forward for in vitro site-directed mutagenesis reactions using the GENEART Site-Directed Mutagenesis System from Thermo Fisher Scientific. To achieve this, two complementary mutagenic oligonucleotide primers were designed (obtained from Sigma-Aldrich) and used to generate gene cDNA expression constructs with desired mutations. Mutations were confirmed using Sanger sequencing, before being delivered into cells using lentiviral infection.

### Plasma DNA sequencing

DNA extraction was performed with QIAmp DNA Mini kit (Qiagen). Library preparation was done with the Oncomine Focus Assay (Thermo Fisher Scientific) following the manufacturer's instructions. After bar coding, libraries were equalized to 100 pM. The sequencing template was prepared using the IonPGMSequencing 200 Kit v2 and sequenced in an Ion Select 318 chip using the PGM Sequencing 200 Kit v2 with 500 flows. Hotspot mutations in 35 genes were targeted using the Oncomine Focus Assay (Thermo Fisher) (Supplemental Table S7). Variant Caller v4.0.r73742 was used for variant calling with the Ion Reporter Software. All filtered variants were also analyzed with the Integrative Genomic Viewer (IGV v2.3) software.

## Data access

All raw sequence data from this study have been submitted to the European Genome-Phenome Archive (EGA; http://www.ebi.ac.uk/ega/), which is hosted at the EBI, under accession numbers EGAS00001001743, EGAS00001001744, and EGAS00001001745. SLAPenrich is implemented as a collection of R scripts and functions and it is publicly available at https://github.com/saezlab/SLAPenrich. The computational pipeline to reproduce the presented results is implemented in the BrammeldEtAl_analysis.R script (also available in the Supplemental Methods).

## Supplementary Material

Supplemental Material
