# Supplementary material for: Genome-wide chemical mutagenesis screens allow unbiased saturation of the cancer genome and identification of drug resistance mutations
Source: Genome Res. 2017 Apr;27(4):613–25. doi: 10.1101/gr.213546.116 (PMC5378179; doi:10.1101/gr.213546.116)
Supplement: Supplemental Material [file supp_27_4_613__index.html]

Genome-wide chemical mutagenesis screens allow unbiased saturation of the cancer genome and identification of drug resistance mutations — Supplemental Material 

# Genome-wide chemical mutagenesis screens allow unbiased saturation of the cancer genome and identification of drug resistance mutations

## Supplemental Material

- Supplemental\_Fig\_S1.pdf
- Supplemental\_Fig\_S2.pdf
- Supplemental\_Fig\_S3.pdf
- Supplemental\_Fig\_S4.pdf
- Supplemental\_Fig\_S5.pdf
- Supplemental\_Fig\_S6.pdf
- Supplemental\_Fig\_S7.pdf
- Supplemental\_Fig\_S8.pdf
- Supplemental\_Fig\_S9.pdf
- Supplemental\_Fig\_S10.pdf
- Supplemental\_Fig\_S11.pdf
- Supplementary\_Methods.docx
- Supplemental\_Table\_S7.xlsx
- Supplemental\_Table\_S8.xlsx
- Supplemental\_Table\_S9.xlsx
- Supplemental\_Table\_S1.xlsx
- Supplemental\_Table\_S4.xlsx
- Supplemental\_Table\_S2.xlsx
- Supplemental\_Table\_S5.xlsx
- Supplemental\_Table\_S3.xlsx
- Supplemental\_Table\_S6.xlsx
- Supplemental\_analysis.R
