# Supplementary material for: Genome-wide chemical mutagenesis screens allow unbiased saturation of the cancer genome and identification of drug resistance mutations
Source: Genome Res. 2017 Apr;27(4):613–25. doi: 10.1101/gr.213546.116 (PMC5378179; doi:10.1101/gr.213546.116)
Supplement: Supplemental Material [file supp_gr.213546.116_Supplemental_Fig_S1.pdf]

Supplemental Figure S1

A

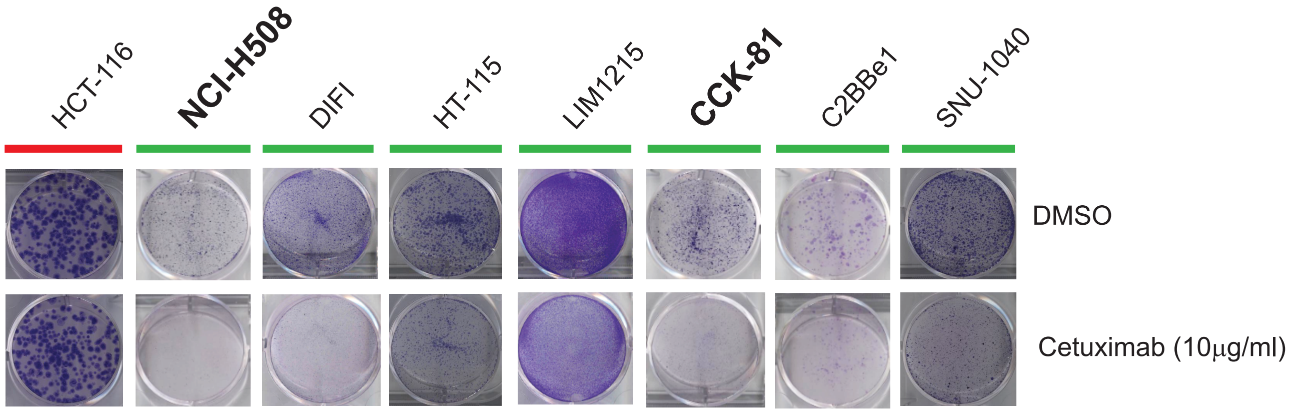

B

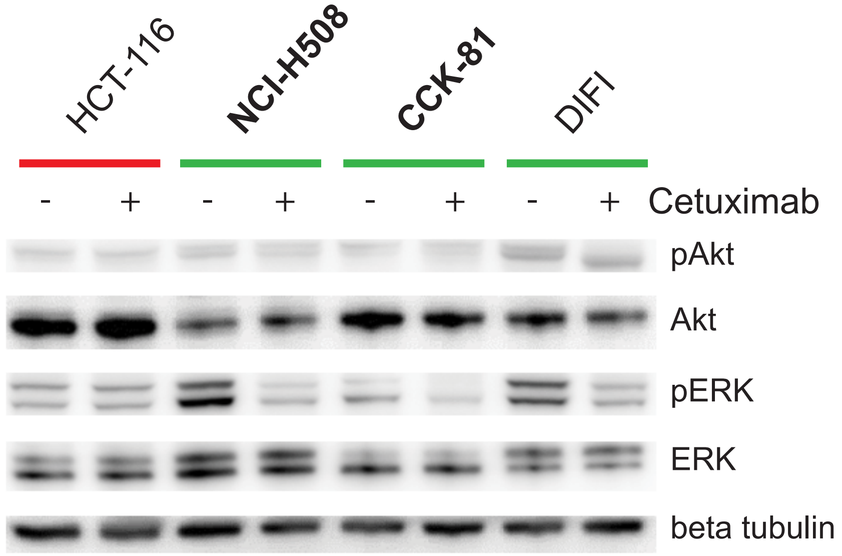

Supp Figure S1. (A) Clonogenic survival assays of 8 KRAS/NRAS/BRAF wild-type colorectal cell lines together with a KRAS G13D mutant line (HCT116) treated with 10µg/ml Cetuximab and assayed at 21 days. Plates were stained with Giemsa. (B) Effect of Cetuximab treatment at 6 hours on MAPK and PI3K signalling in Cetuximab sensitive (green bar) versus resistant (red bar) colorectal cell lines. Beta tubulin is used as a loading control.
