# Supplementary material for: Genome-wide chemical mutagenesis screens allow unbiased saturation of the cancer genome and identification of drug resistance mutations
Source: Genome Res. 2017 Apr;27(4):613–25. doi: 10.1101/gr.213546.116 (PMC5378179; doi:10.1101/gr.213546.116)
Supplement: Supplemental Material [file supp_gr.213546.116_Supplemental_Fig_S2.pdf]

Supplemental Figure S2

NCI-H508 MSS clones

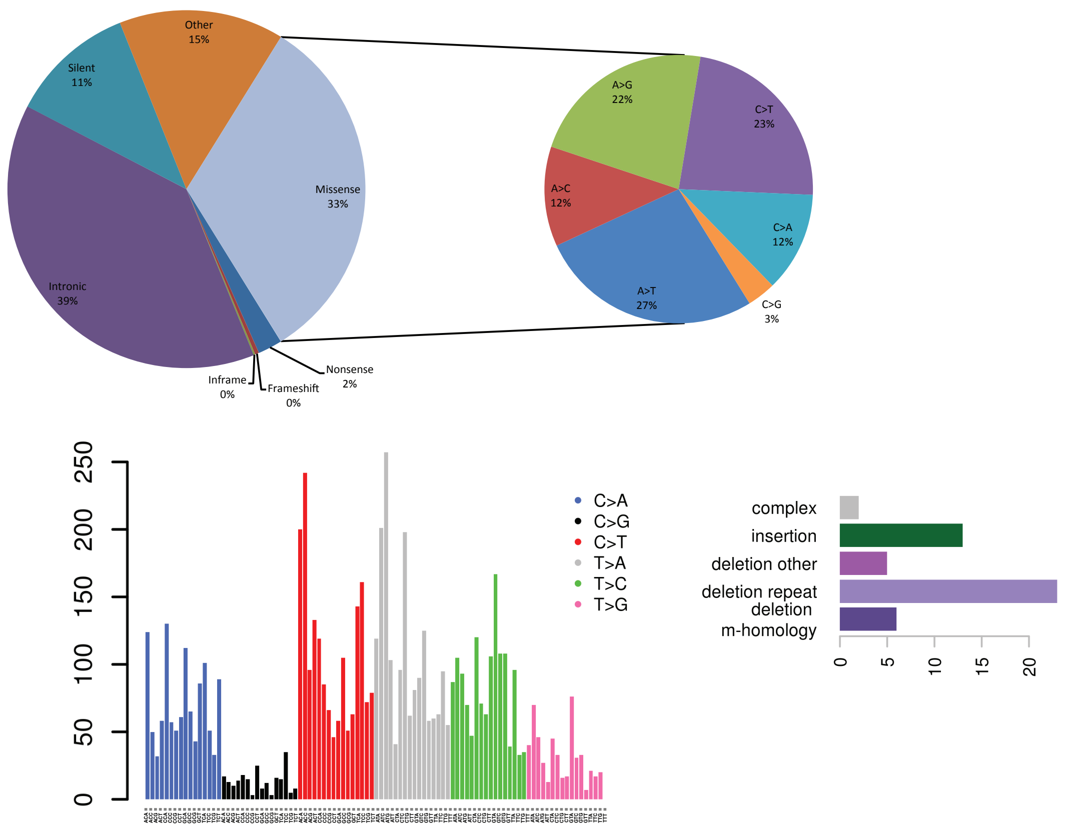

CCK-81 MSI clones

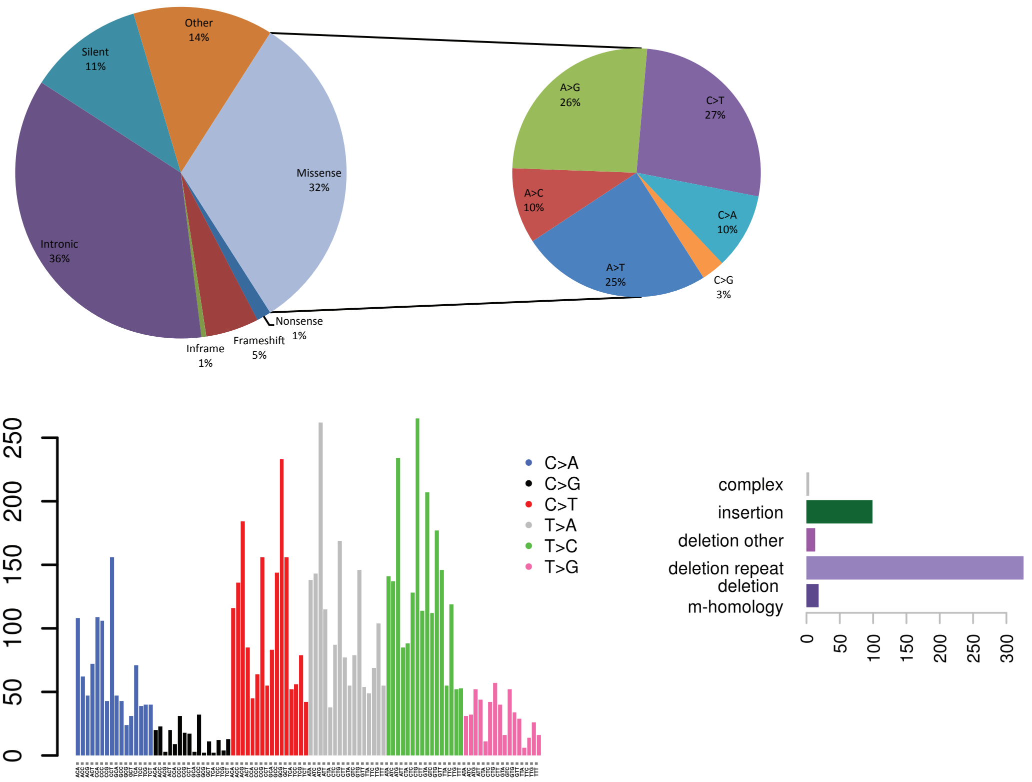

Supp Figure S2. Mutational spectrum of ENU-derived Cetuximab-resistant clones in the NCI-H508 and the CCK-81 cell lines. The proportion of mutation types (left hand pie chart) and the missense mutations represented by the 6 classes of base substitutions (C>T, C>A, C>G, A>T, A>C, A>G) (right-hand pie chart) is displayed for drug-resistant clones from NCI-H508 and CCK-81 cell lines.The trinucleotide representation of the 6 base substitution types (left panel) and the pattern of indels (right panel) is displayed below.
