# Supplementary material for: Genome-wide chemical mutagenesis screens allow unbiased saturation of the cancer genome and identification of drug resistance mutations
Source: Genome Res. 2017 Apr;27(4):613–25. doi: 10.1101/gr.213546.116 (PMC5378179; doi:10.1101/gr.213546.116)
Supplement: Supplemental Material [file supp_gr.213546.116_Supplemental_Fig_S3.pdf]

Supplemental Figure S3

NCI-H508 clones

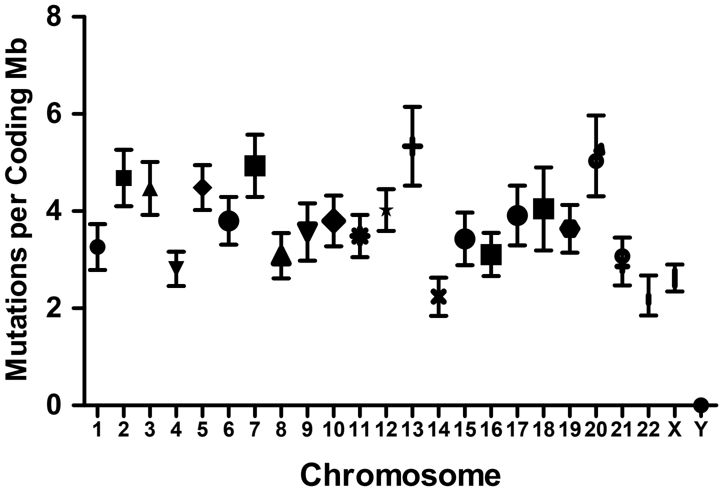

CCK-81 clones

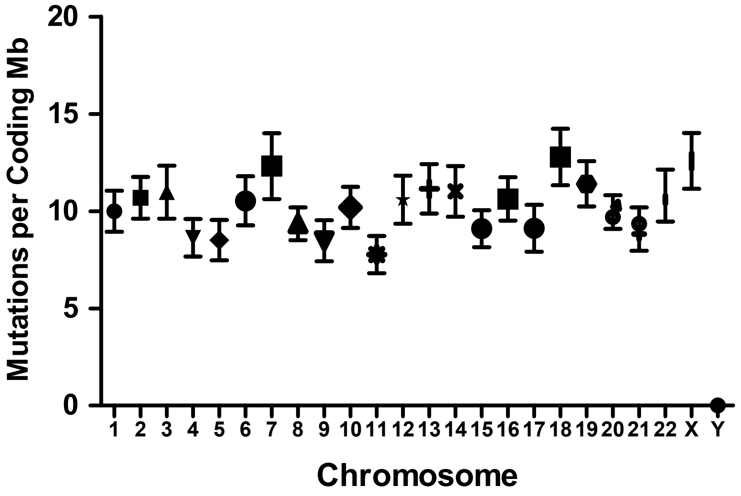

Supp Figure S3. Distribution of coding mutations per chromosome. All mutations detected in the NCI-H508 and CCK-81 cetuximab-resistant clones were combined and the mean number of mutations calculated per Mb of coding exon per chromosome.
