# Supplementary material for: Genome-wide chemical mutagenesis screens allow unbiased saturation of the cancer genome and identification of drug resistance mutations
Source: Genome Res. 2017 Apr;27(4):613–25. doi: 10.1101/gr.213546.116 (PMC5378179; doi:10.1101/gr.213546.116)
Supplement: Supplemental Material [file supp_gr.213546.116_Supplemental_Fig_S4.pdf]

Supplemental Figure S4

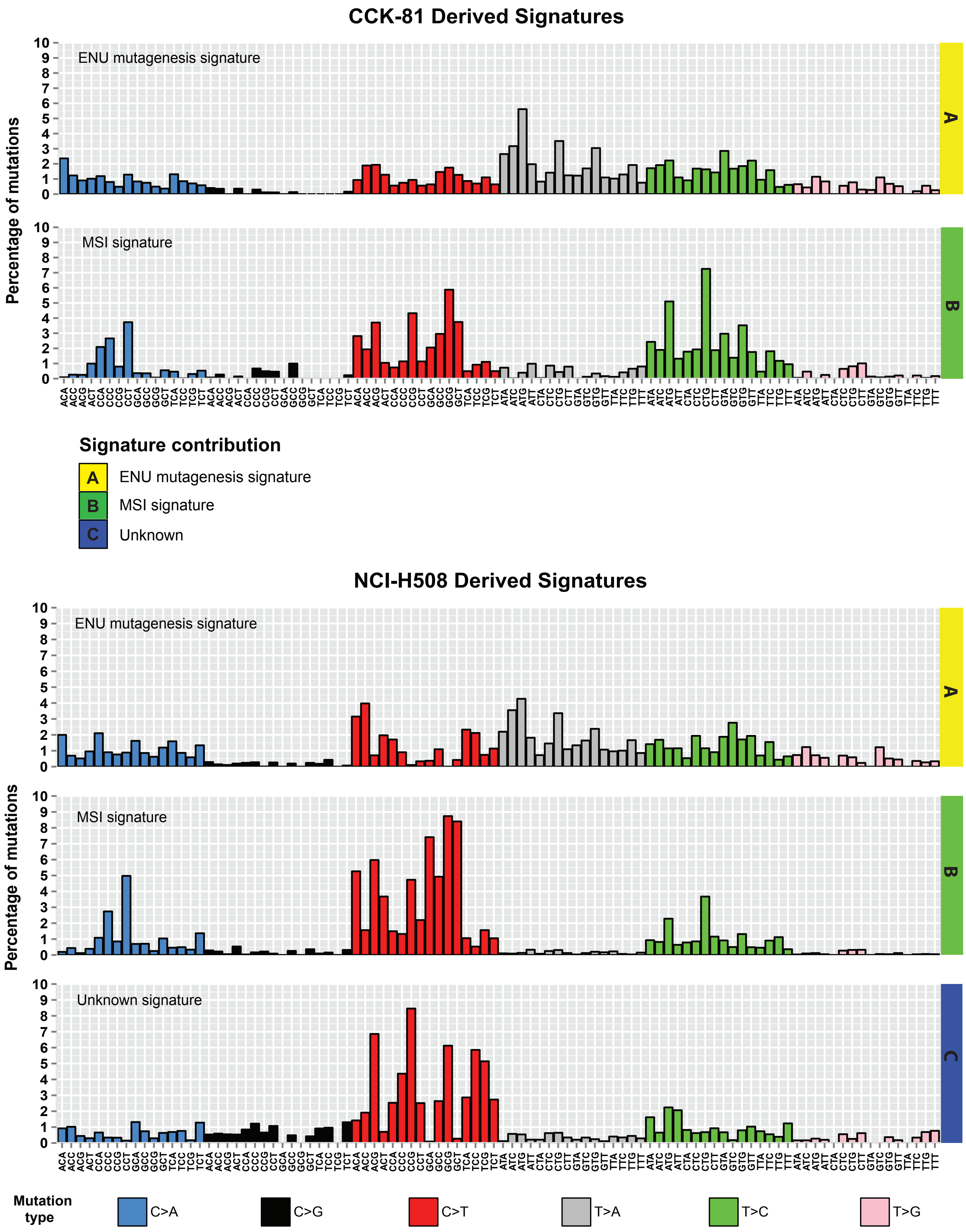

Supp Figure S4. Mutational signatures extracted from ENU-derived CCK-81 and NCI-H508-Cetuximab resistant clones based on the trinucleotide pattern of substitutions and using for each locus the substitution class (C>T, C>A, C>G, A>T, A>C, A>G) and the sequence context immediately 5' and 3' to the mutated base. Signature A is that of the ENU-derived mutations whereas Signature B is that of MSI (microsatellite instability). Signature C is of unknown aetiology.
