# Supplementary material for: Genome-wide chemical mutagenesis screens allow unbiased saturation of the cancer genome and identification of drug resistance mutations
Source: Genome Res. 2017 Apr;27(4):613–25. doi: 10.1101/gr.213546.116 (PMC5378179; doi:10.1101/gr.213546.116)
Supplement: Supplemental Material [file supp_gr.213546.116_Supplemental_Fig_S5.pdf]

Supplemental Figure S5

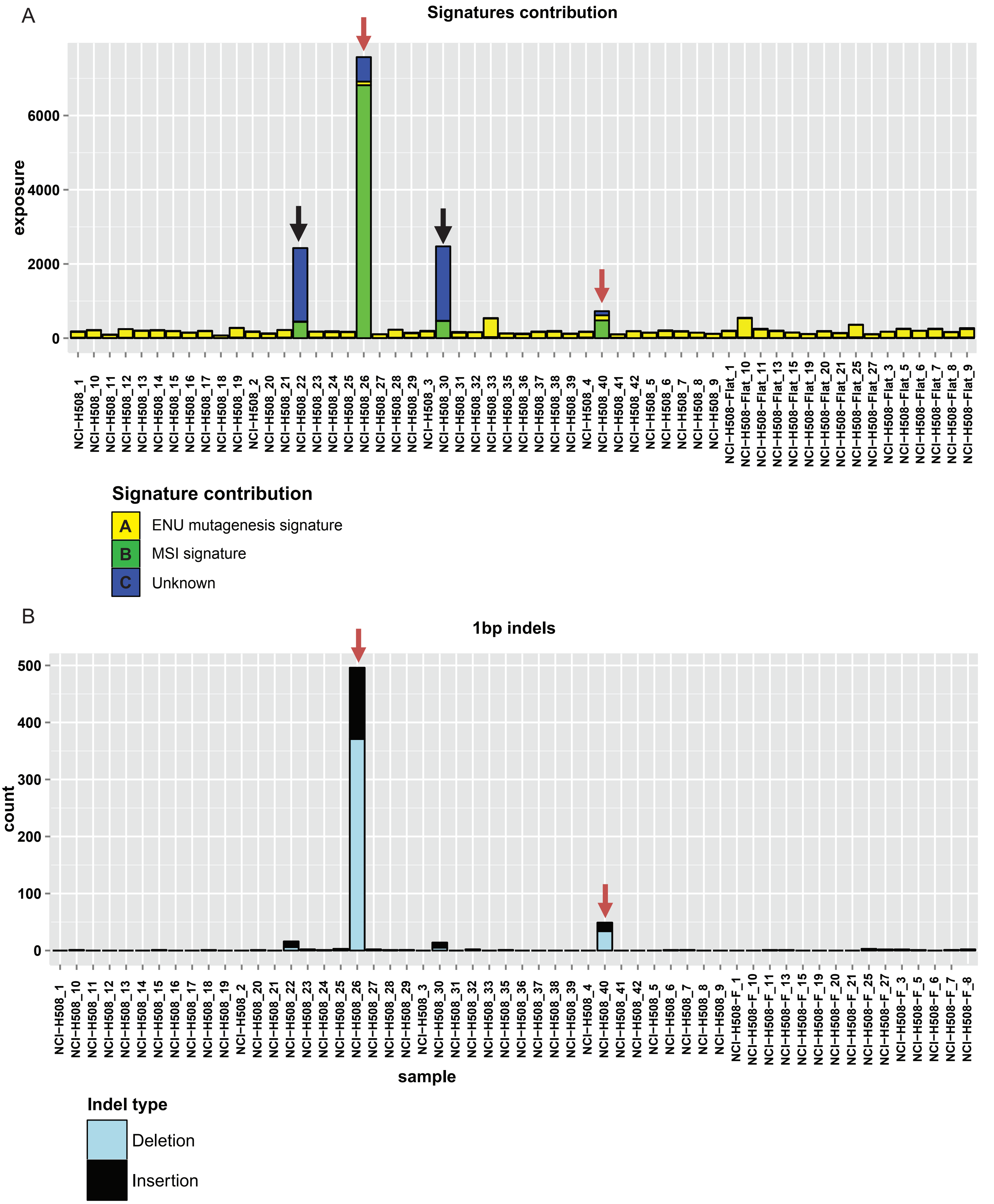

Supp Figure S5. The contribution from each of the NCI-H508-derived Cetuximab resistant clones towards generating Signatures A, B and C. (A) Total number of mutations with contributions from corresponding mutational signatures. (B) Total number of 1bp insertions and deletions with respective contributions.
