# Supplementary material for: Genome-wide chemical mutagenesis screens allow unbiased saturation of the cancer genome and identification of drug resistance mutations
Source: Genome Res. 2017 Apr;27(4):613–25. doi: 10.1101/gr.213546.116 (PMC5378179; doi:10.1101/gr.213546.116)
Supplement: Supplemental Material [file supp_gr.213546.116_Supplemental_Fig_S6.pdf]

Supplemental Figure S6

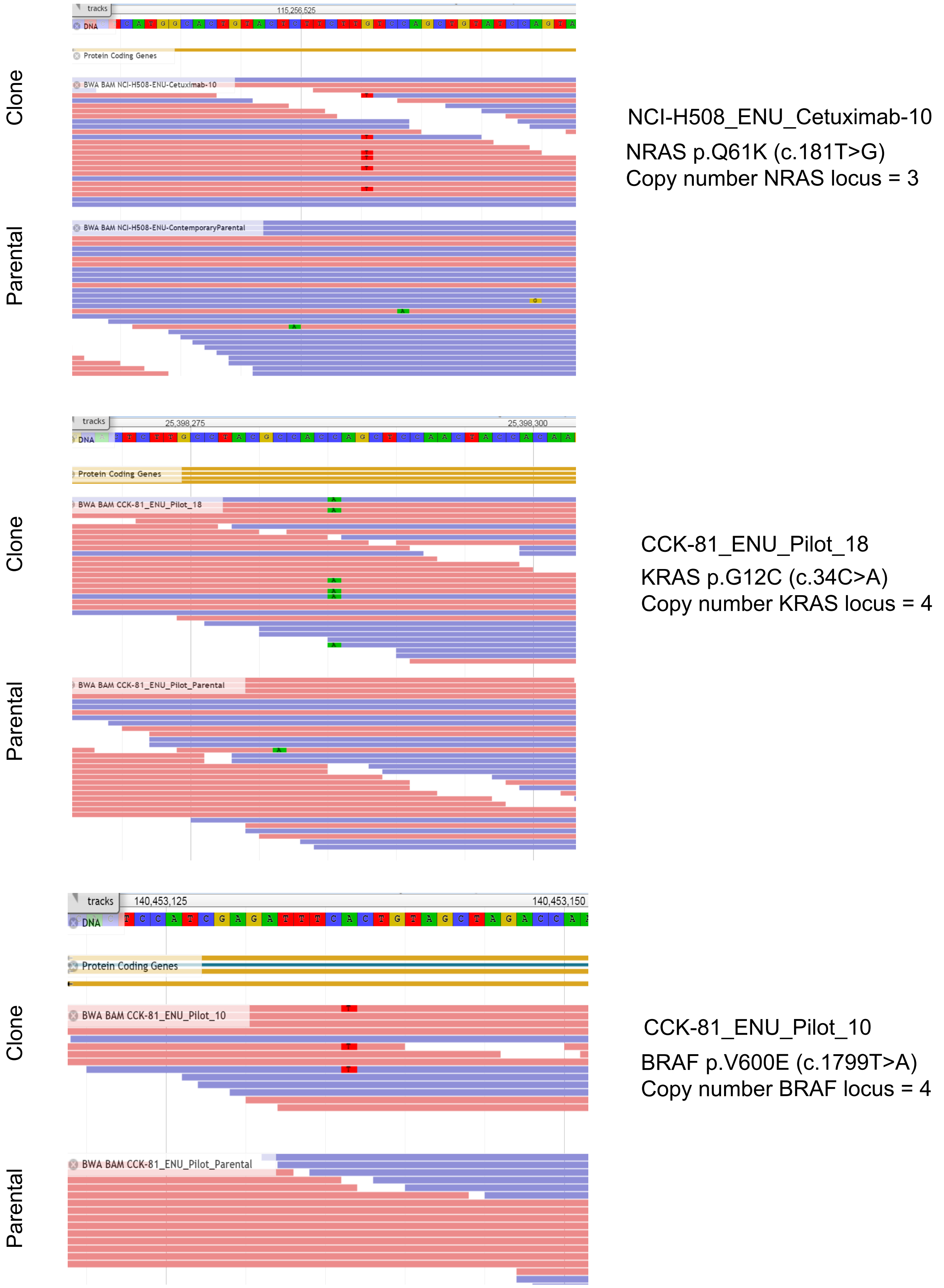

Supp Figure S6. Whole exome sequence visualisation of BRAF, NRAS and KRAS mutations in both CCK-81 and NCI-H508-derived Cetuximab resistant clones. These are supported by bidirectional reads which are absent from matched contemporary parental sequences. Forward and reverse reads are shown in red and blue respectively. Reported bases differing from the reference sequence are shown in red. The sequence of the Cetuximab-resistant clone is shown above that of the corresponding contemporary parental sequence. Genome coordinates and nucleotide sequence of a canonical transcript of the gene are shown at top of figure. Note that the ratio of mutant to wild-type reads in each of the clones above is dependent on the total number of alleles and where we assume that only one allele is likely to be mutated. The copy number for each gene is indicated adjacent to each figure.
