# Supplementary material for: Genome-wide chemical mutagenesis screens allow unbiased saturation of the cancer genome and identification of drug resistance mutations
Source: Genome Res. 2017 Apr;27(4):613–25. doi: 10.1101/gr.213546.116 (PMC5378179; doi:10.1101/gr.213546.116)
Supplement: Supplemental Material [file supp_gr.213546.116_Supplemental_Fig_S7.pdf]

Supplemental Figure S7

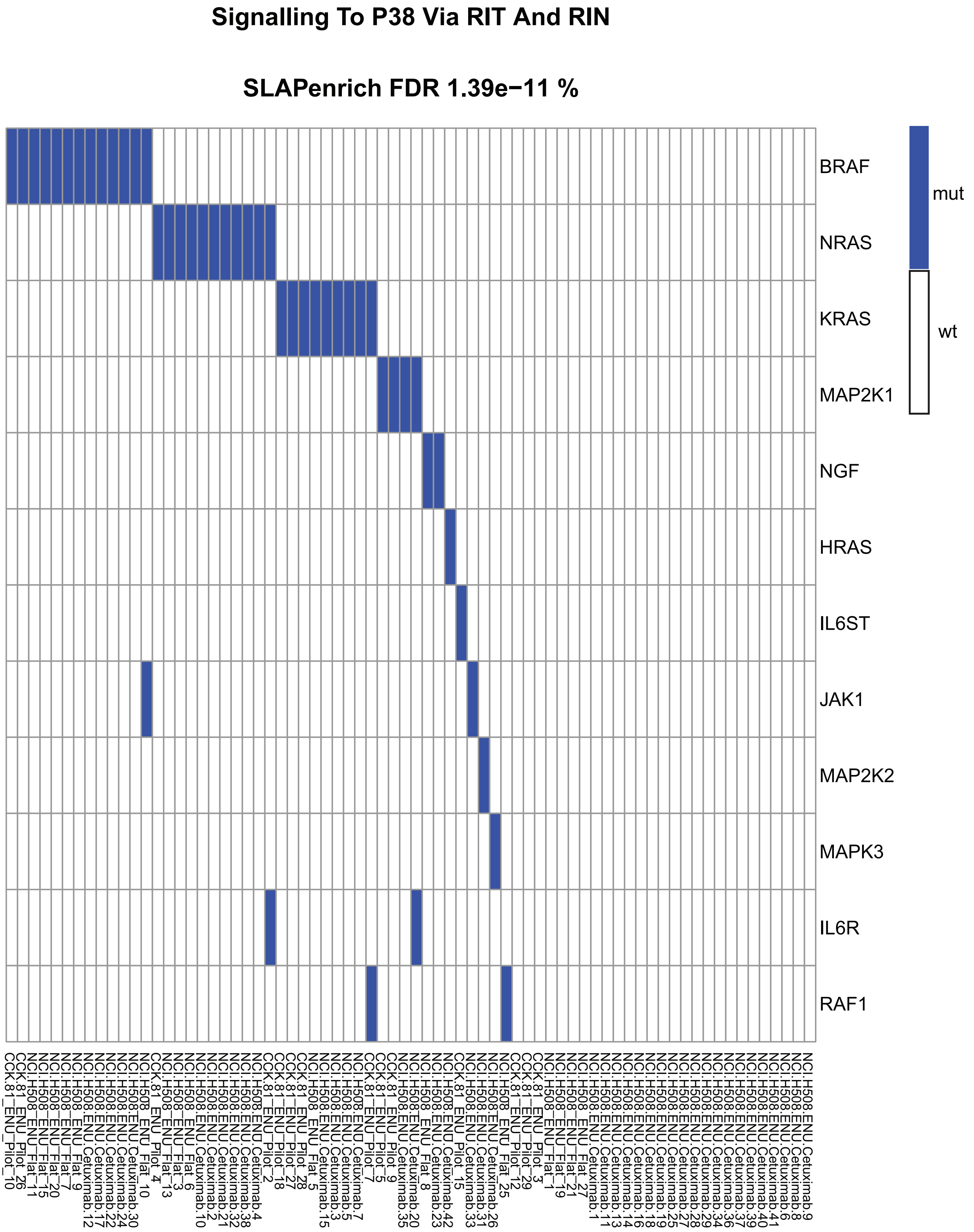

Supp Figure S7. Heatmap of all mutant ENU clones and mutated genes found in the most significantly enriched network following analysis of data by SLAP-Enrich. Clones are arrayed along the bottom of the figure and mutations are grouped together by gene as well as by pattern of mutual exclusivity.
