# Supplementary material for: Genome-wide chemical mutagenesis screens allow unbiased saturation of the cancer genome and identification of drug resistance mutations
Source: Genome Res. 2017 Apr;27(4):613–25. doi: 10.1101/gr.213546.116 (PMC5378179; doi:10.1101/gr.213546.116)
Supplement: Supplemental Material [file supp_gr.213546.116_Supplemental_Fig_S8.pdf]

Supplemental Figure S8

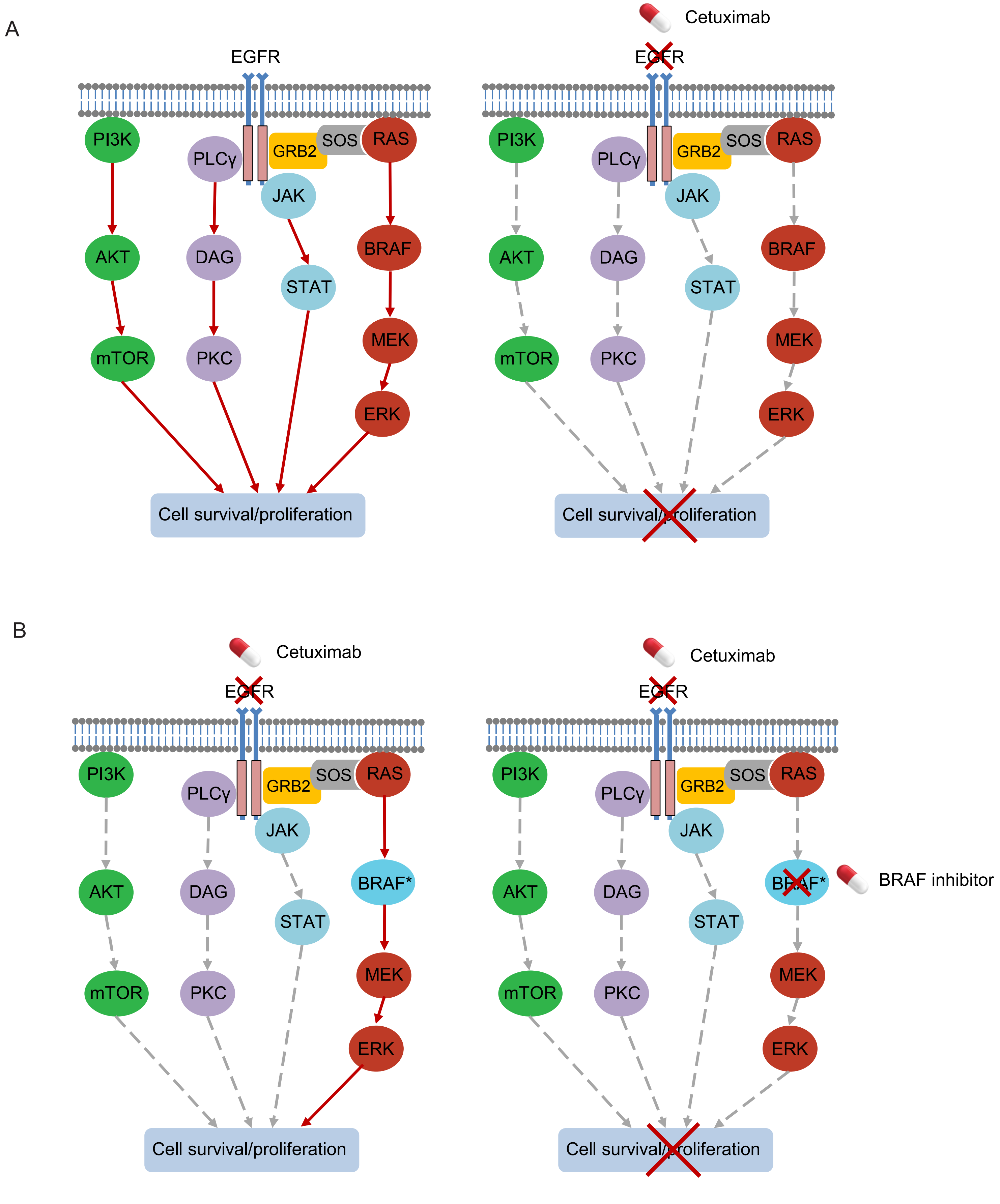

Supp Figure S8. (A) In the parental cells, blockade of EGFR signaling inhibits a variety of downstream signaling pathways, including PI3K, JAK-STAT, PLC-PKC and MAPK. (B) In a Cetuximab-resistant clone that has acquired an activating mutation in BRAF (asterisk), treatment of the cells with Cetuximab fails to suppress MAPK signaling and cells continue to proliferate. Only when a BRAF inhibitor is combined with Cetuximab are all the relevant signaling pathways inhibited and cell survival/proliferation reduced.
