# Supplementary material for: Genome-wide chemical mutagenesis screens allow unbiased saturation of the cancer genome and identification of drug resistance mutations
Source: Genome Res. 2017 Apr;27(4):613–25. doi: 10.1101/gr.213546.116 (PMC5378179; doi:10.1101/gr.213546.116)
Supplement: Supplemental Material [file supp_gr.213546.116_Supplemental_Fig_S9.pdf]

Supplemental Figure S9

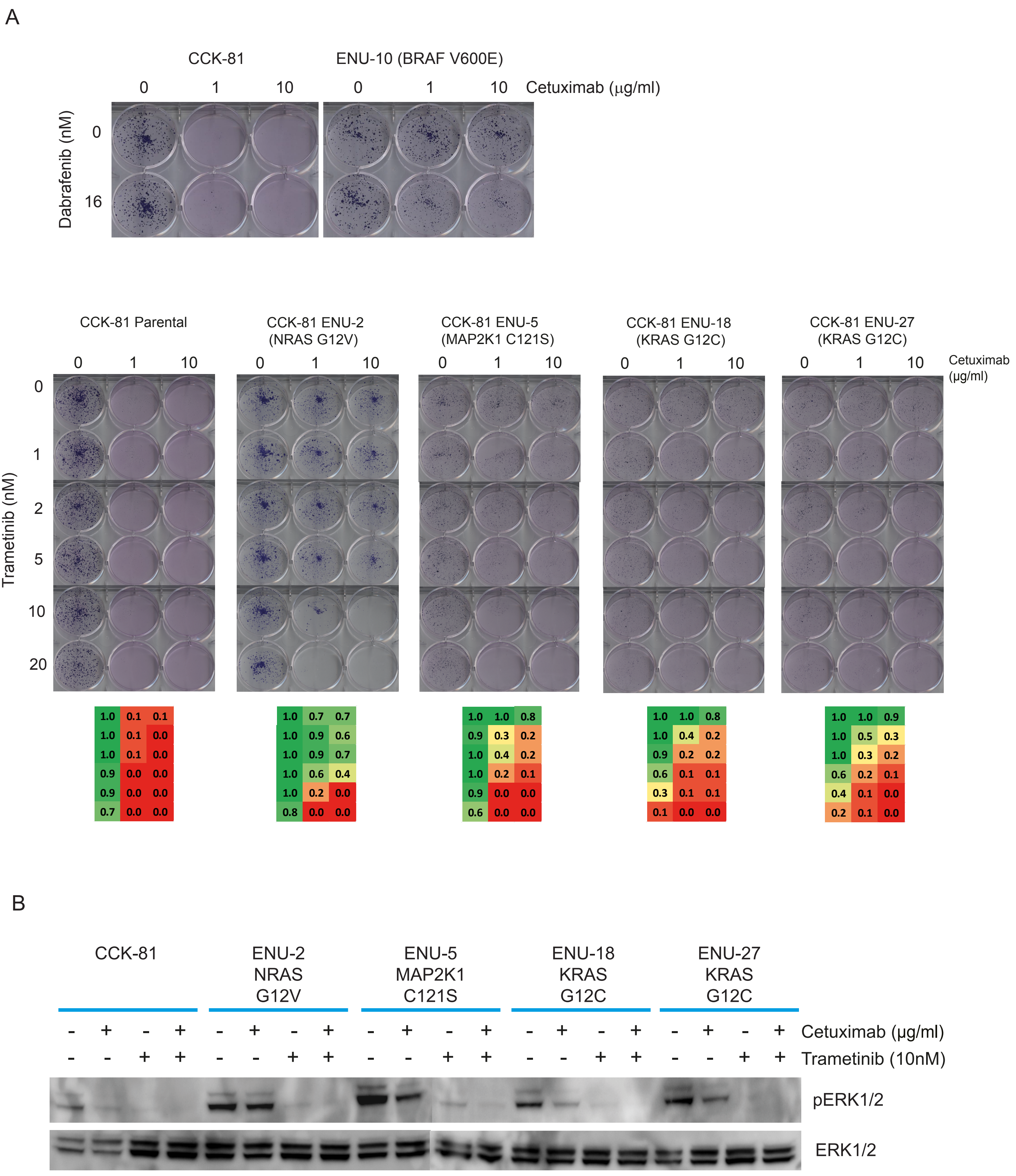

Supp Figure S9. (A) Re-sensitization of Cetuximab resistant cells by targeting MAPK signalling. Clonogenic survival assays at 21 days in the CCK-81 parental cell line and 3 Cetuximab-resistant clones from the ENU screen (ENU-10, ENU-5 and ENU-18) treated with Cetuximab (0, 1 or 10μg/ml) combined with the BRAF inhibitor Dabrafenib (16nM) or the MEK1 inhibitor Trametinib (1-20nM). The ENU-derived resistance mutation detected in each resistant clone is indicated in brackets. Below each figure is a heatmap of the viability normalised to each control well. (B) Immunoblot of CCK-81 cell line and mutant ENU clones (mutation indicated for each clone) treated with Cetuximab (10μg/ml), Trametinib (10nM) or a combination of both for 3 hours and pERK/total ERK assayed.
