# Supplementary material for: Genome-wide chemical mutagenesis screens allow unbiased saturation of the cancer genome and identification of drug resistance mutations
Source: Genome Res. 2017 Apr;27(4):613–25. doi: 10.1101/gr.213546.116 (PMC5378179; doi:10.1101/gr.213546.116)
Supplement: Supplemental Material [file supp_gr.213546.116_Supplemental_Fig_S10.pdf]

Supplemental Figure S10

A

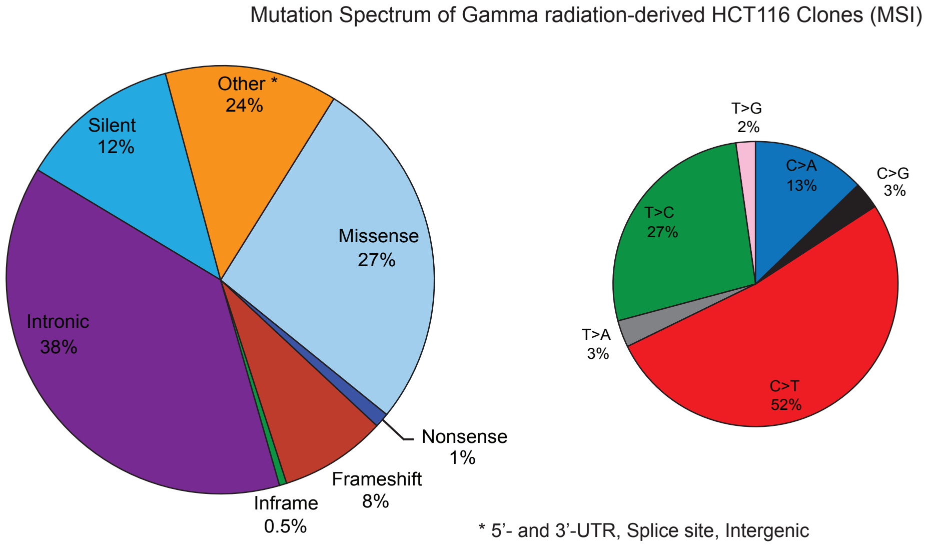

B

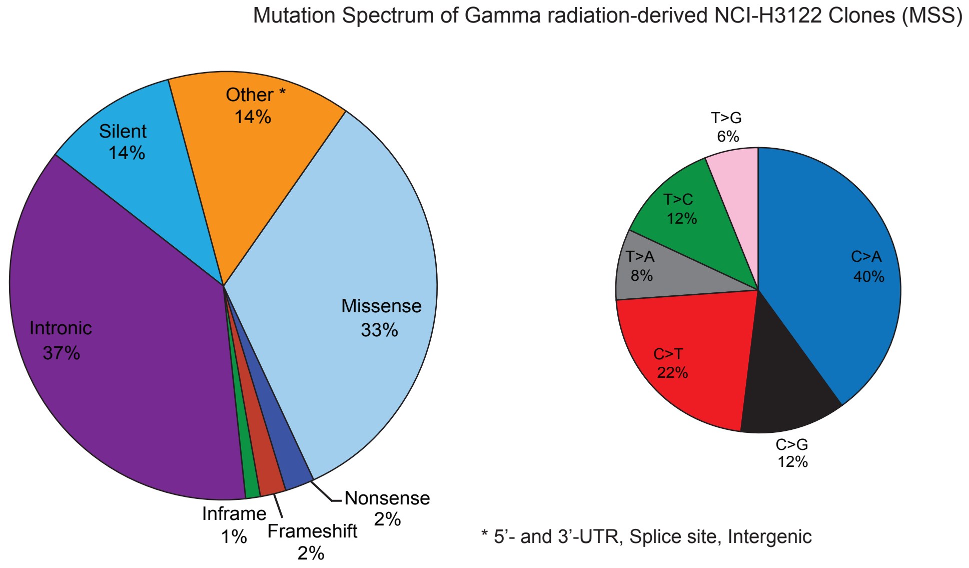

Supp Figure S10. Mutation spectrum and types of mutations observed following whole exome sequencing of 9 single cell-derived clones from the (A) MSI HCT116 cell line and the (B) MSS NCI-H3122 cell line. Each cell line was gamma irradited with 1Gy or 10Gy and the following day seeded as single cells in order to generated single cell-derived colonies.
