# Supplementary material for: Genome-wide chemical mutagenesis screens allow unbiased saturation of the cancer genome and identification of drug resistance mutations
Source: Genome Res. 2017 Apr;27(4):613–25. doi: 10.1101/gr.213546.116 (PMC5378179; doi:10.1101/gr.213546.116)
Supplement: Supplemental Material [file supp_gr.213546.116_Supplemental_Fig_S11.pdf]

Supplemental Figure S11

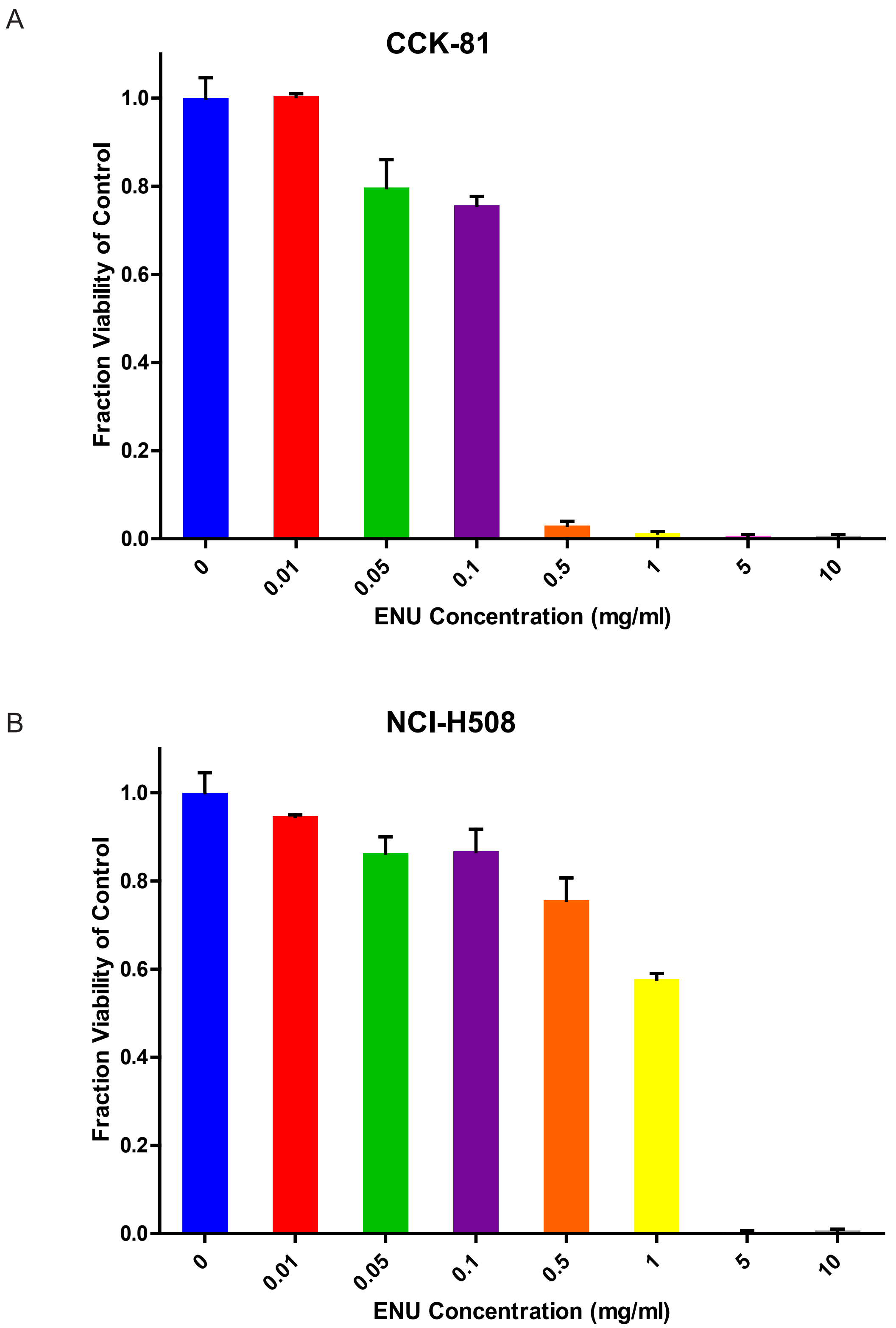

Supp Figure S11. Cetuximab sensitive colorectal cancer cell lines show variable sensitivity to ENU. (A) CCK-81 and (B) NCI-H508 cell viability after incubation with ENU for 24 hours. Cells were assayed using cell titre blue after a 24 hour recovery period without ENU. Experiments were performed in triplicate. Error bars represent the standard deviation of triplicate values.
