# Supplementary material for: Genome-wide chemical mutagenesis screens allow unbiased saturation of the cancer genome and identification of drug resistance mutations
Source: Genome Res. 2017 Apr;27(4):613–25. doi: 10.1101/gr.213546.116 (PMC5378179; doi:10.1101/gr.213546.116)
Supplement: Supplemental Material [file supp_gr.213546.116_Supplementary_Methods.docx]

**Supplementary Methods**

**Sample Level Analysis of Pathway Enrichments (SLAP-Enrich)**

*Pathway gene-sets collection and post-processing.*

A collection of pathway gene sets was downloaded from the Pathway Commons data portal (v4-201311) (<http://www.pathwaycommons.org/archives/PC2/v4-201311/>) and used in SLAP-Enrich. This contained an initial catalogue of 2,893 gene sets (one for each pathway) assembled from multiple public available resources, such as Reactome, Panther, HumanCyc and PID, and covering 8,148 unique genes. From this catalogue, gene sets containing less than 4 genes were discarded.

In order to remove redundancies those gene sets 1) corresponding to the same pathway across different resources or 2) with a large overlap (Jaccard index (JI) > 0.8, as detailed below) were merged together by intersecting them. The gene sets resulting from this compression were then added to the collection (with a joint pathway label) and those participating in at least one merging were discarded. The final collection resulting from this pre-processing was composed by 1,636 gene sets, for a total amount of 8,056 unique genes. Given two gene sets $P_{1}$ and $P_{2}$ the corresponding JI is defined as:

$$JI\left( P_{1}, P_{2} \right)=\frac{\left| P_{1} \cap P_{2} \right|}{\left| P_{1} \cup P_{2} \right|}$$

## *Statistical framework.*

## Let $\boldsymbol{G}\boldsymbol{=}\left\{ \boldsymbol{g}_{\boldsymbol{1}}\boldsymbol{,}\boldsymbol{g}_{\boldsymbol{2}}\boldsymbol{,\cdots,}\boldsymbol{g}_{\boldsymbol{n}} \right\}$ be a list including all the genes whose mutational status across a set of samples $\boldsymbol{S}\boldsymbol{=}\left\{ \boldsymbol{s}_{\boldsymbol{1}}\boldsymbol{,}\boldsymbol{s}_{\boldsymbol{2}}\boldsymbol{,\cdots,}\boldsymbol{s}_{\boldsymbol{m}} \right\}$ has been determined by whole exome sequencing profiling, and $\boldsymbol{f:G}\boldsymbol{\times}\boldsymbol{S}\boldsymbol{\to}\left\{ \boldsymbol{0}\boldsymbol{, 1} \right\}$ a function defined as $\boldsymbol{f}\left( \boldsymbol{g}\boldsymbol{,}\boldsymbol{s}_{\boldsymbol{i}} \right)\boldsymbol{=}\left\{ \boldsymbol{1} \text{if }\boldsymbol{g} \text{is altered in sample }\boldsymbol{s}_{\boldsymbol{i}} \text{and 0 otherwise} \right\}$.

Given a pathway gene set $P$, the aim is to assess whether there is a tendency for that pathway to be recurrently genetically altered across the samples in $S$. In what follows we will refer to $P$ and the pathway corresponding to $P$ interchangeably.

We assume that the pathway $P$ is altered in sample $s_{i}$ if $\exists g\in P$ such that $g\in G$ and $f\left( g,s_{i} \right)=1$, i.e. at least one gene in the pathway $P$ is altered in the $i$-th sample. First of all we quantify how likely it is to observe at least one gene belonging to $P$ altered in sample $s_{i}$. To quantify this probability, let us introduce the variable $X_{i}=\left| \left\{ g\in P:g\in G \text{and }f\left( g, s_{i} \right)=1 \right\} \right|$, whose value is equal to the number of genes in $P$ that are altered in sample $s_{i}$.

Under the assumption of both a gene-wise and sample-wise statistical independence, the probability of $X_{i}$ assuming a value greater or equal than 1 is given by:

Equation 1

$$p_{i}=\text{Pr}\left( X_{i}\geq1 \right)=1-\exp\left( -\rho k \right),$$

where $k=\sum_{g\in P} \mathcal{L(}g)$ is the sum of the exonic block length of all the genes in pathway $P$, and $\rho$ the background mutation rate, which can be computed from analysed dataset directly or set to established estimated values (typically ${10}^{-6}/\text{n}\text{ucleotide}$) [Other possible models to computed these individual probabilities are implemented in our public available code].

The $p_{i}$ can be considered as the success probability of a set of Bernoulli trials $\left\{ i \right\}$ (with $i=1, \ldots, M$) and summing them across all the elements of $S$ gives the expected number of samples harbouring a mutation in at least one gene belonging to the pathway $P$:

Equation 2

$$E\left( P \right)= \sum_{i=1}^{M} p_{i}.$$

On the other hand, if we consider a function $\phi$ on the domain of the $X$ variables, defined as $\phi\left( X \right)= \left\{ 1 \text{if }X\geq1 \text{and }0 \text{otherwise} \right\}$, then summing the values assumed by this function across all the samples $S$ gives the observed number of samples harbouring a mutation in at least one gene belonging to $P$:

$$O\left( P \right)= \sum_{i=1}^{M} \phi\left( X_{i} \right).$$

A pathway alteration index quantifying the deviance of $O\left( P \right)$ from its expectation can be then computed as:

$$\mathcal{A}\left( P \right)=\log_{10} \frac{O(P)}{E(P)}.$$

To assess the significance of such deviation, let us observe that the probability of the event $O\left( P \right)=y$, with $y\leq M$, (i.e. the probability of observing exactly $y$ samples in which the pathway $P$ is altered) distributes as a Poisson binomial $B$ (a discrete probability distribution modeling the sum of a set of independent Bernoulli trials that are not identically distributed). In our case, the $i$-th Bernoulli trial accounts for the event “the pathway $P$ is altered in the sample $s_{i}$” and its probability of success is given by $p_{i}$ introduced above. The parameters of such $\mathcal{B}$ distribution are then the probabilities $\left\{ p_{i} \right\}$, and its mean is given by Equation 2.

The probability of the event $O\left( P \right)=y$ can be written then as:

$$\text{Pr}\text{(}O\left( P \right)=y\mathcal{)=B}\left( \rho,y \right)=\sum_{A\in F_{y}} \prod_{i\in A} p_{i}\prod_{j\in A^{c}} (i-p_{j})$$

where $F_{y}$ is the set of all the possible subsets of $y$ that can be selected from $\left\{ 1, 2, \ldots, M \right\}$ (for example if $M=3$, then $F_{2}=\left\{ \left\{ 1, 2 \right\},\left\{ 1, 3 \right\},\left\{ 2, 3 \right\} \right\}$), and $A^{c}$ is the complement of $A$ (i.e. $\left\{ 1, 2, \ldots, M \right\}\backslash A).$

Hence a p-value can be computed against the null hypothesis that $O\left( P \right)$ is drawn from a Poisson binomial distribution parametrised through the vector of probabilities $\left\{ p_{i} \right\}.$ Such p-value can be derived for an observation $O\left( P \right)=z$, with $z\leq M$, as:

$$\text{Pr}\left( O\left( P \right)\geq z \right)=\sum_{i=z}^{M} \text{Pr}(O\left( P \right)=i)\text{=} \sum_{i=z}^{M} \mathcal{B(}\left\{ p_{i} \right\},i).$$

## *Mutual Exclusivity Filter*

## After correcting the p-values yielded by testing all the pathways in the collection with the Benjamini-Hockberg method (<http://www.math.tau.ac.il/~ybenja/MyPapers/benjamini_hochberg1995.pdf>), SLAP-Enrich further filters the pathways whose enrichment false discovery rate (FDR) is below a user defined threshold (in this study 5%) based on mutual exclusivity criteria as a further evidence of positive selection. Particularly for a given enriched pathway $\boldsymbol{P}$, an *exclusive coverage* score $\boldsymbol{M(}\boldsymbol{P)}$ is computed as

$$M\left( P \right)= 100 \frac{\dot{O}(P)}{O(P)}$$

where $O(P)$ is the number of samples in which at least one gene belonging to the pathway gene-set $P$ is mutated, and $\dot{O}(P)$ is the number of samples in which exactly one gene belonging to the pathway gene-set $P$ is mutated. In this study, all the pathways $P$ with $M\left( P \right)\geq50$ pass this final filter.

*R-Code to reproduce the presented results*

All the code and data objects needed to reproduce the results presented in our manuscript are public available at: <https://github.com/saezlab/SLAPenrich>. All the instructions needed to reproduce our results are included in the BrammeldEtAl_analysis.R script, also reported below.

## The SLAPenrich R package is available at github.com/saezlab/SLAPenrich/blob/master/SLAPenrich_0.1.tgz

library(SLAPenrich)

#Loading the data object containing the genome-wide exonic content block lenghts

data(SLAPE.all_genes_exonic_content_block_lengths_ensemble)

#Loading the data object containing genome-wide recent official Hugo gene symbols and synonyms

data(SLAPE.hgnc.table)

#Loading the data object containing the reference pathway gene-set collection pre-processed for redundancy reduction

data(SLAPE.PATHCOM_HUMAN_nr_i_hu_2014)

#Reading the set of variants (included in Supplementary Table 6, which must be saved in csv format)

#filename must be updated with the local path to this variant file

variants<-

SLAPE.readDataset(filename = 'BrammeldEtAl_variants.csv')

#Checking/updating gene symbols

Dataset<-

SLAPE.check_and_fix_gs_Dataset(variants,updated.hgnc.table = hgnc.table)

#Performing a SLAPenrich analysis

PFPw<-

SLAPE.analyse(EM = Dataset,PATH_COLLECTION = PATHCOM_HUMAN,

show_progress = TRUE,

NSAMPLES = 2,

NGENES = 2,

accExLength = TRUE,

BACKGROUNDpopulation = rownames(Dataset),

path_probability = 'Bernoulli',

GeneLenghts = GECOBLenghts)

#Saving results in a csv file

SLAPE.write.table(PFP = PFPw,EM = Dataset,

filename = "./SLAPEresults.csv",

fdrth=5,exclcovth = 50,PATH_COLLECTION = PATHCOM_HUMAN,GeneLenghts = GECOBLenghts)

#Producing pdf with enriched pathways' visualisations

SLAPE.serialPathVis(EM = Dataset,PFP = PFPw,fdrth = 5,exCovTh = 50,

PATH = "./",PATH_COLLECTION = PATHCOM_HUMAN)

**Identification of drug resistance genes based on the impact of coding mutations**

To identify recurrently mutated driver genes we used a dN/dS method that considers the mutation spectrum, the sequence of each gene, the impact of coding substitutions (synonymous, missense, nonsense, splice site) and the variation of the mutation rate across genes, based on the one described in Greenman et al, 2006 (Greenman et al. 2006), extended to consider the variation of the mutation rate across genes using covariates, inspired by Lawrence et al., 2013 (Lawrence et al. 2013).

Each mutation type is modelled as a Poisson distributed random variable, with a rate given by a product of the mutation rate and the impact of selection. We used 192 mutation parameters to describe the different mutability of each trinucleotide in the coding strand (*e.g.* A**C**G>A**T**G), thus accounting for any context-dependent mutagenesis from one base up and downstream and transcriptional strand bias in mutation rates. Three selection parameters measure the observed-over-expected ratio of missense (*ω_mis_*), nonsense (*ω_non_*) and essential splice site (*ω_spl_*) mutations. Essential splice sites were defined as positions -2 and -1 upstream of an exon start and positions +1, +2 and +5 downstream of an exon end(Greenman et al. 2006). For example, the expected number of synonymous A**C**G>A**T**G mutations is modelled as:

*λ_syn,ACG>ATG_* = *t* * *r_ACG>ATG_* * *S_syn,ACG>ATG_*

And the expected number of missense A**C**G>A**T**G mutations as:

*λ_mis,ACG>ATG_* = *t* * *r_ACG>ATG_* * *S_mis,ACG>ATG_ * ω_mis_*

The rate parameter *r_ACG>ATG_* refers to the relative rate of A**C**G>A**T**G transitions per ACG site in the sequence. *t* represents the local mutation rate normalised by sequence composition. The inclusion of *t* makes one of the substitution rates unnecessary and so we fix *r_TTT>TGT_* = 1. *ω_mis_* represents the rate of missense mutations relative to synonymous mutations, and can be interpreted as the observed-over-expected ratio of missense mutations. Values of *ω* significantly higher than 1 reflect dominant positive selection and values lower than 1 dominant negative selection. *S_mis,ACG>ATG_* represents the number of sites that can suffer a missense A**C**G>A**T**G mutation in the sequence of the gene (thus accounting for the length and sequence composition of the gene).

The likelihood of observing a given number of missense A**C**G>A**T**G mutations in a particular gene (*n_mis,ACG>ATG_*), given the expected rate (*λ_mis,ACG>ATG_*), is calculated as follows:

L_mis,ACG>ATG_ = Pois(*λ_mis,ACG>ATG_* | *n_mis,ACG>ATG_*) = *λ^n^* * e^-^*^λ^*/*n*!

The joint likelihood of the entire model is the product of the individual likelihoods (or the sum of log-likelihoods) from each of these equations.

L_gene_ = Π_j∈_ _{1,2,…,192}_ [Pois(*λ_syn,j,gene_*|*n_syn,j,gene_*)*Pois(*λ_mis,j,gene_*|*n_mis,j,gene_*)*Pois(*λ_non,j,gene_*|*n_non,j,gene_*)*Pois(λ*_spl,j,gene_*|*n_spl,j,gene_*)]

This allows us to quantify separately the strength of selection at missense, nonsense and essential splice site mutations, while avoiding the confounding effects of gene length, sequence composition and different rates of each substitution type. Maximum-likelihood estimates and confidence intervals for the 195 parameters are obtained using Poisson regression. To obtain accurate estimates of the 192 rate parameters, they are estimated from the entire collection of mutations. These 192 rates are then assumed constant across genes and gene-specific maximum-likelihood estimates for *t*, *ω_mis_*, *ω_non_* and *ω_spl_* are obtained for each gene. For every gene, a likelihood ratio test with three degrees of freedom is performed comparing the likelihood of the unconstrained model (free values for *ω_mis_*, *ω_non_* and *ω_spl_*), to the neutral model (*ω_mis_*=*ω_non_*=*ω_spl_*=1), yielding a *P*-value for each gene. This allows the detection of driver genes, evolving under positive selection.

The method explained above allows every gene to have a different background mutation rate (*t* can take any value), which makes the method very conservative when none or few synonymous mutations per gene are available. We have described and used this method before to detect driver genes on large datasets (Wong et al. 2014).

We now extend this method to explicitly model the variation of the mutation rate across all genes to increase sensitivity on smaller datasets. If we assume that the background mutation rate (*t_gene_*) varies across genes and that this variation can be described by a Gamma distribution (Γ) (mean=*μ*, shape=*θ*), the new joint likelihood of the model (L_gene_’) can be expressed as:

L_gene_’ = Γ(*t_gene_*|*θ,μ_gene_*) * L_gene_

Where Γ represents the probability of a gene having a specific background mutation rate (*t_gene_*), given the (Gamma) distribution of mutation rates across genes. To estimate this Gamma distribution we use a negative binomial regression. Briefly, the number of synonymous mutations per gene is the observed variable, the expected number of mutations in the gene assuming uniform mutation rates across genes (*E_syn,gene_* = Σ_j_ *λ_syn,j,gene_*) is the offset and as covariates we use the default covariates in *MutSigCV*: HiC, replication time and the log of the expression level (Lawrence et al. 2013). Although the method does not require covariates, good covariates reduce the unexplained variation of the mutation rate across genes (which manifests as a lower overdispersion, or what is the same, as higher values of *θ*), in turn increasing the power to detect genes under selection. We performed the negative binomial regression using the *glm.nb* function of the *MASS* R package.

model = glm.nb(formula = *n_syn_* ~ offset(log(*E_syn_*)) + log(*expression*) + *reptime* + *hic* )

The negative binomial regression yields an expected rate, *μ_gene_*, for every gene (the expected background mutation rate *t* of the gene given its covariates) and an overdispersion parameter *θ* (describing how much do mutation rates deviate from the mean *μ_gene_*). Note that, using the Gamma-Poisson mixture interpretation of the negative binomial distribution; this approach treats the observed number of synonymous mutations per gene as Poisson observations from an underlying Gamma distribution of mutation rates across genes.

The addition of the Gamma distribution to the likelihood function probabilistically constrains the values of the background mutation rate (*t_gene_*) when fitting the null (neutral) and alternative (selection) models, making this method much more powerful in small and medium-size datasets. For very large datasets, with many synonymous mutations per gene, the mutation rate of every gene would be mainly determined by its synonymous mutations, and so the method converges to the one without the Gamma constraint.

*Driver analysis for coding indels*

The dN/dS method described above is restricted to single nucleotide substitutions, for which synonymous substitutions can be used to estimate the background mutation rate. Owing to the lack of a neutral reference for the indel rate in coding sequences, here we used a different approach.

Let *n_indel,gene_* be the number of indels in a gene and *S_indel,gene_* the size of the coding region of the gene. To minimise the impact of recurrent artefacts, multiple identical indels in different samples were only counted once, with *n_indels_* then representing the number of individual indel sites per gene across all samples. The average density of unique indels per base-pair across all genes is: *μ_indel_* = Σ_gene_(*n_indel,gene_*) / Σ_gene_(*S_indel,gene_*), and the expected number of indels per gene: *E_indel,gene_* = *μ_indel_* * *S_indel,gene_*. To quantify the variation of the indel rate across genes we used a negative binomial regression of the form: glm.nb(formula = *n_indel_* ~ offset(log(*E_indel_*)) -1). We did not include covariates as they did not seem to improve the fit substantially. This treats *n_indel,gene_* as Poisson observations with rates *λ_indel,gene_*, where *λ_indel,gene_* are gamma distributed across genes.

To minimize the impact of driver events inflating the over-dispersion of the model, we excluded from the fit any gene in the *a priori* list of putative driver genes. Driver events in other genes would make this approach conservative, but it should still be able to detect strong deviations from the background distribution. The observed counts for each gene (*n_indel,gene_*) are compared to the background distribution using a negative binomial test, with the overdispersion parameter (*θ*) estimated by the negative binomial regression, yielding *P*-values for indel recurrence for each gene.

*Combined analysis for indels and substitutions*

To detect genes under significant selective pressure by either point mutations or indels, for each gene the *P*-values from the dN/dS analysis of substitutions and from the recurrence analysis of indels were combined using Fisher’s method. Multiple testing correction (Benjamini-Hochberg FDR) was performed for all genes, stratifying the FDR correction to increase sensitivity (as described in Sun *et al*. 2006 (Sun et al. 2006)). To achieve a low false discovery rate a conservative q-value cut-off of <0.05 was used for significance, and considered significant any gene with qmis_sfdr<0.05 OR qglobal_sfdr<0.05.

**References**

Greenman C, Wooster R, Futreal PA, Stratton MR, Easton DF. 2006. Statistical analysis of pathogenicity of somatic mutations in cancer. *Genetics* **173**: 2187-2198.

Lawrence MS, Stojanov P, Polak P, Kryukov GV, Cibulskis K, Sivachenko A, Carter SL, Stewart C, Mermel CH, Roberts SA et al. 2013. Mutational heterogeneity in cancer and the search for new cancer-associated genes. *Nature* **499**: 214-218.

Sun L, Craiu RV, Paterson AD, Bull SB. 2006. Stratified false discovery control for large-scale hypothesis testing with application to genome-wide association studies. *Genetic epidemiology* **30**: 519-530.

Wong CC, Martincorena I, Rust AG, Rashid M, Alifrangis C, Alexandrov LB, Tiffen JC, Kober C, Chronic Myeloid Disorders Working Group of the International Cancer Genome C, Green AR et al. 2014. Inactivating CUX1 mutations promote tumorigenesis. *Nature genetics* **46**: 33-38.
