# Supplementary material for: Genome-wide chemical mutagenesis screens allow unbiased saturation of the cancer genome and identification of drug resistance mutations
Source: Genome Res. 2017 Apr;27(4):613–25. doi: 10.1101/gr.213546.116 (PMC5378179; doi:10.1101/gr.213546.116)
Supplement: Supplemental Material [file supp_gr.213546.116_Supplemental_analysis.R.html]

Supplemental\_analysis 

# Genome-wide chemical mutagenesis screens allow unbiased saturation of the cancer genome and identification of drug resistance mutations
